# A Six‐Electron Energy Storage Material for Ultra‐Stable Aqueous Organic Redox Flow Batteries

**DOI:** 10.1002/advs.202514452

**Published:** 2025-09-30

**Authors:** Xiaowei Zhang, Lu Li, Yunlong Ji, Pan Wang

**Affiliations:** ^1^ Department of Chemistry Zhejiang University Hangzhou Zhejiang 310058 China; ^2^ Key Laboratory of Precise Synthesis of Functional Molecules of Zhejiang Province Department of Chemistry School of Science and Research Center for Industries of the Future Westlake University Hangzhou 310030 China; ^3^ School of Chemistry and Materials Science Hangzhou Institute for Advanced Study University of Chinese Academy of Sciences 1 Sub‐lane Xiangshan Hangzhou 310024 China; ^4^ Division of Solar Energy Conversion and Catalysis at Westlake University Zhejiang Baima Lake Laboratory Co., Ltd. Hangzhou Zhejiang 310000 China

**Keywords:** aqueous organic redox flow batteries (AORFBs), energy storage, multiple‐electron transfer, negative electrolyte, phenazine derivatives

## Abstract

Aqueous organic redox flow batteries (AORFBs) offer sustainable, large‐scale energy storage using tunable, earth‐abundant organic molecules, avoiding resource limitations. While most aqueous redox flow battery materials utilize single‐ (n = 1) or dual‐electron (n = 2) transfer mechanisms, stable multi‐electron (n > 4) redox systems remain largely unexplored. A six‐electron (n = 6) phenazine‐based negative electrolyte, 2,2′,2′'‐(diquinoxalino[2,3‐a:2′,3′‐c]phenazine‐2,8,14‐triyltris(oxy))tripropionic acid (PPA) is engineered with a *π*‐extended fused‐ring core and branched hydrophilic side chains. As supported by molecular dynamics simulation, the C─O linked tri‐substituted propanoic acid groups in PPA disrupt intermolecular *π*–*π* stacking, achieving an unprecedented aqueous solubility of 1.2 m with six electron storage (7.2 m electron concentration, 193.0 Ah L^−1^ theoretical capacity). When paired with a ferrocyanide, the PPA‐based AORFB gives a cell voltage of 1.31 V at 3.0 m electron concentration, along with a Coulombic efficiency exceeding 99.8% and a 85% utilization of its six‐electron capacity. The system demonstrates exceptional cycling stability, sustaining a capacity decay rate of 0.032% per cycle, 0.095% per day over 80 operational days. This work provides a scalable approach for stable multi‐electron energy storage materials applicable in AORFBs.

## Graphical abstract

A six‐electron phenazine‐based negative electrolyte **PPA** is engineered with a *π*‐extended core and branched hydrophilic chains, achieving high solubility and chemical stability. Being used as a negative electrolyte when paired with ferrocyanide, it enables an aqueous organic redox flow battery (AORFB) with high capacity, exceptional cycling stability, and minimal capacity decay over thousands of cycles.

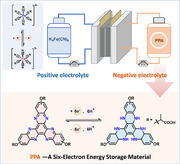

## 1 Introduction

The utilization of electricity derived from renewable energy sources is increasingly regarded as a critical strategy to address escalating global energy demand and urgent environmental crises. Aqueous redox flow batteries (ARFBs) have emerged as a promising technology for integrating renewable energy and stabilizing the storage capacity of electrical grids, owing to their decoupled power and energy density, superior energy conversion efficiency, and extended operational lifespan.^[^ ^1^, ^2^, ^3^ ^]^ While vanadium‐based ARFBs have been a predominant focus in flow battery research due to their unique single‐element electrolyte architecture, their commercial viability is constrained by vanadium supply chain limitations and price volatility.^[^ ^4^, ^5^ ^]^ Aqueous organic redox flow batteries (AORFBs) employ earth‐abundant organic redox‐active molecules with synthetically tunable properties through molecular engineering, offering a sustainable alternative without relying on limited resources.^[^ ^6^, ^7^ ^]^

Energy density, a pivotal performance metric for battery applicability, is determined by cell voltage and volumetric capacity of the electrolyte in an AORFB system. Cell voltage depends on the redox potential difference between the positive electrolyte (posolyte) and negative electrolyte (negolyte). The volumetric capacity is determined by active material solubility and effective electron concentration. Current strategies to improve the solubility, including functional groups incorporating, molecular symmetry breaking, and counterion effect, etc., have yielded different families of energy storage materials for AORFB applications, such as single‐electron (n = 1) transfer dominating in ferrocenes,^[^ ^8^, ^9^, ^10^ ^]^ nitroxides,^[^ ^11^, ^12^ ^]^ azobenzene^[^ ^13^ ^]^ and viologens,^[^ ^14^, ^15^, ^16^, ^17^, ^18^ ^]^ while quinones^[^ ^19^, ^20^, ^21^, ^22^ ^]^ and phenazines^[^ ^23^, ^24^, ^25^ ^]^ enabling stable two‐electron (n = 2) redox cycling. Another effective strategy is molecular architecture optimization by introducing multiple redox‐active moieties within one single molecule without compromising solubility. Redox‐active energy storage molecules capable of more than two electron transfers (n >2) have been recently reported, with advancements of four electron transfer posolytes (n = 4), such as CSFA^[^ ^26^ ^]^ and PTO‐PTS.^[^ ^27^ ^]^ However, degradation pathways from redox reactions remain a critical challenge. Notably, stable multi‐electron transfer negolytes are still lacking.

Phenazines have shown particular promise as stable negolytes by fine‐tuning their molecular orbitals, enabling tailored redox activity and stability.^[^ ^28^, ^29^, ^30^, ^31^ ^]^ Building on their reversible redox‐active aromatic skeletons, the development of multi‐electron negolytes could unlock high‐energy‐density AORFBs. A viable strategy involves integrating multiple redox‐active moieties into a single conjugated molecular framework to boost electron concentration while maintaining atomic economy. However, this approach frequently compromises solubility, as evidenced by previously reported systems (TPyTz)Cl_6_ with four electron transfer (n = 4), TPz‐2, and HATNTA bearing six‐electron transfer capabilities (n = 6).^[^ ^32^, ^33^, ^34^ ^]^ For example, TPz‐2 achieves 0.3 m solubility in 1.0 m NaOH, yet its 0.005 m cell suffers severe capacity decay of 0.6% per cycle, 55% per day over 50 cycles. In contrast, HATNTA exhibits improved solubility of 0.58 m in 1.5 m KOH and stability, with a 0.25 m cell showing a reduced capacity decay of 0.168% per day in long‐term cycling for 10 days. Despite these advances, critical gaps persist for practical applications with both solubility and cell cycling for long‐term stability, posing significant challenges to further development. The central challenge lies in the synergistic optimization of the effective electron transfer concentration, aqueous solubility while preserving its desired low redox potentials for negolytes, and robust chemical stability with multi‐step redox reversibility.

**Figure 1:**
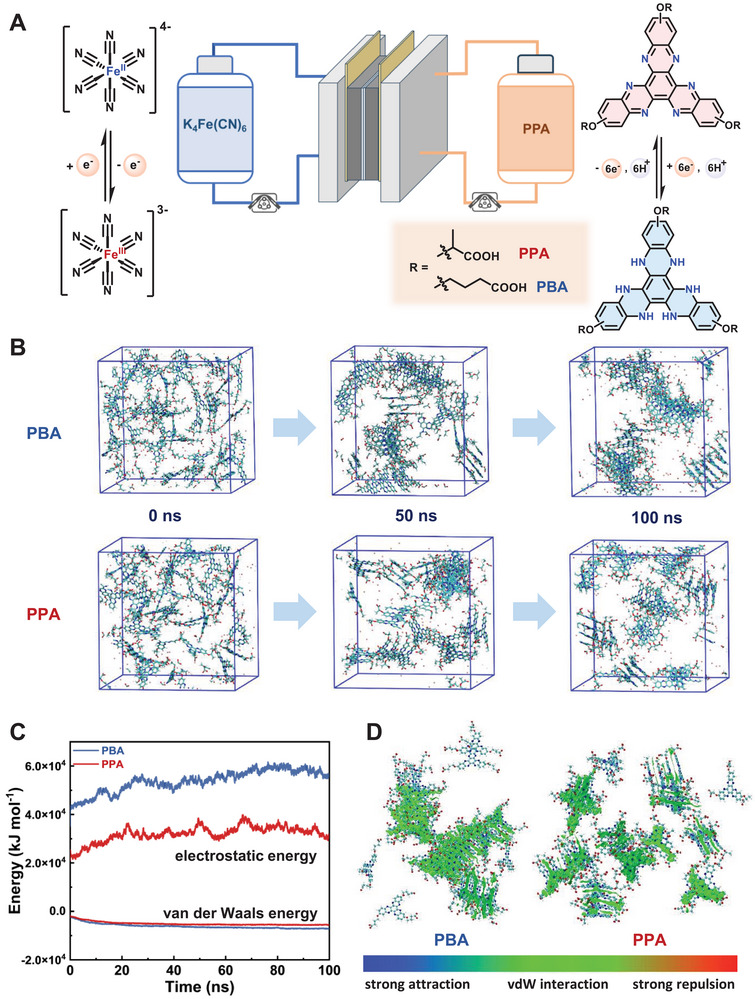
A) Schematic diagram of representative flow cell assembled with **PPA** paired with K_4_Fe(CN)_6_. B) Molecular dynamics simulations snapshots of 300 mm **PBA** and **PPA** in H_2_O. Snapshots of the front view of **PBA** and **PPA** for 0, 50, and 100 ns represent removed water molecules. C) The comparison of electrostatic energy and van der Waals energy of **PBA** and **PPA**. D) Visual analysis of weak interactions between molecules of **PPA** and **PBA**, while the green zone is the area where van der Waals forces are effective.

Herein, we developed a six‐electron storage molecule 2,2′,2′'‐(diquinoxalino[2,3‐a:2′,3′‐c]phenazine‐2,8,14‐triyltris(oxy))tripropionic acid and its isomers (**PPA**), possessing a π‐extended fused‐ring conjugation with branched hydrophilic functionalities. The carbon–oxygen (C–O) linked branched propanoic acid side chains disrupt intermolecular *π*–*π* stacking, promoting the aqueous solubility to 7.2 m electron concentration, i.e., 193.0 Ah L^−1^ theoretical volumetric capacity and lowering redox potential to −0.77 V vs standard hydrogen electrode (SHE). When paired with K_4_Fe(CN)_6_/K_3_Fe(CN)_6_ as the posolyte, the **PPA**‐based AORFB demonstrates a cell voltage of 1.31 V at 3.0 m electron concentration. The battery system achieves an average Coulombic efficiency (CE) exceeding 99.8% and an 85% capacity utilization of six electron capacity, maintaining exceptional cycling stability with a capacity decay rate of 0.032% per cycle, 0.095% per day over 80 operational days (**Figure** **1**A). This molecular engineering strategy, balancing electron density and solubility through functionalization, offers a generalizable framework for other multi‐electron energy storage materials.

## 2 Discussion

**PPA** and its regio‐isomers, bearing branched propanoic acid functional groups, were synthesized via a two‐step synthesis with a condensation reaction of methyl 2‐(3,4‐diaminophenoxy)propanoate and cyclohexanehexone, followed by an ester hydrolysis reaction, giving **PPA** as a yellow solid in 78% overall yield (Scheme S1, Supporting Information). For comparative studies, a linear butyric acid functionalized analog 4,4′,4′'‐(diquinoxalino[2,3‐*a*:2′,3′‐c]phenazine‐2,8,14‐triyltris(oxy))tributyric acid (**PBA**) with regio‐isomers were synthesized under the same synthetic protocol. The non‐hydrolyzed ester precursors of both **PPA** and **PBA** give unresolved signals in ^1^H and ^13^C nuclear magnetic resonance (NMR) spectra, precluding definitive determination of isomer ratios. Liquid chromatography‐mass spectrometry (LC‐MS) analysis of these precursors showed single peaks in chromatograms (Figure S1, Supporting Information), suggesting either chromatographically indistinguishable isomers or dominant formation of one regio‐isomer. For simplicity,** PPA **and **PBA** are used to denote the products, inclusive of potential regio‐isomers.

Preliminary characterizations reveal that **PBA** exhibits an aqueous solubility of 0.4 m in 1.0 m KOH, while the branched carboxylic group functionalized **PPA** significantly promotes its solubility to 0.8 m in 1.0 m KOH, 1.1 m in 1.0 m KCl, and 1.2 m in H_2_O (Figures S2–S5, Supporting Information; **Table** **1**). This solubility enhancement probably arises from the tri‐substituted branched functional groups breaking molecular symmetry and introducing steric interactions, which effectively suppress *π*–*π* stacking between molecules. These experimental observations align with density functional theory (DFT) calculations of solvation thermodynamics, with **PPA** demonstrating a lower Gibbs solvation energy of ΔG_solv_ = −283.86 kcal mol^−1^ (Table S1, Supporting Information).

**Table 1:** Summary of the physicochemical and electrochemical properties of **PBA** and **PPA**.

Compound	E_1/2_ [V vs SHE]^a)^	*k_0_* [cm s^−1^]^b)^	*D* [cm^2^ s^−1^]^c)^	Solubility/ Electron conc. [mol L^−1^]
**PBA** ^d)^	−0.68, −0.48	N.D.^e)^	N.D.^e)^	0.4/2.4 (in 1.0 m KOH)
**PPA** ^d)^	−0.77, −0.58	6.01 × 10^−5^ 1.29 × 10^−4^	1.31 × 10^−7^ 1.65 × 10^−7^	0.8/4.8 (in 1.0 m KOH) 1.1/6.6 (in 1.0 m KCl) 1.2/7.2 (in H_2_O)

^a)^ Redox potential of PBA in 2.0 m KOH, while PPA in 1.0 M KOH;

^b)^ Electron‐transfer rate constant;

^c)^ Diffusion coefficient;

^d)^ **PBA** and **PPA** are in their potassium salts;

^e)^ “N.D.” means not detected.

Molecular dynamics (MD) simulations were further employed to systematically investigate the intermolecular *π*–*π* stacking behavior of **PBA** and **PPA** in solution. Comparative analysis of the simulation snapshots at 0 and 100 ns reveals distinct *π*–*π* stacking between the molecules (Figure 1B; Figure S6, Supporting Information). Specifically, **PBA** forms ordered multilayer aggregates, while **PPA** exhibits only localized stacking and limited aggregation, which could be attributed to the steric hindrance imposed by its branched methyl substituents. Detailed interaction analysis provided quantitative insights into the distinct aggregation behaviors of **PBA** and **PPA**. In the interaction energy analysis, **PBA** exhibits a more negative van der Waals interaction energy compared to **PPA** because of its enhanced π‐π stacking interactions, consistent with its stronger tendency toward molecular aggregation. While both molecules exhibit attractive intermolecular interactions overall, the stronger van der Waals forces in **PBA**, driven by enhanced π‐π stacking, lead to tighter molecular aggregation. At these reduced distances, the Coulombic repulsion between the negatively charged deprotonated groups becomes dominant, resulting in a more positive Coulombic interaction energy for **PBA** that exceeds that of **PPA** (Figure 1C). Intermolecular weak interactions were analyzed from final simulation snapshots using the Independent Gradient Model (IGM) with an isosurface value of 0.005 a.u. (Figure 1D). The IGM visualization reveals significantly more extensive green isosurfaces between **PBA** molecules than in **PPA**, indicating stronger van der Waals interactions. These observations show how branched methyl substituents influence stacking behavior and intermolecular interaction landscapes through side‐chain engineering.

**Figure 2:**
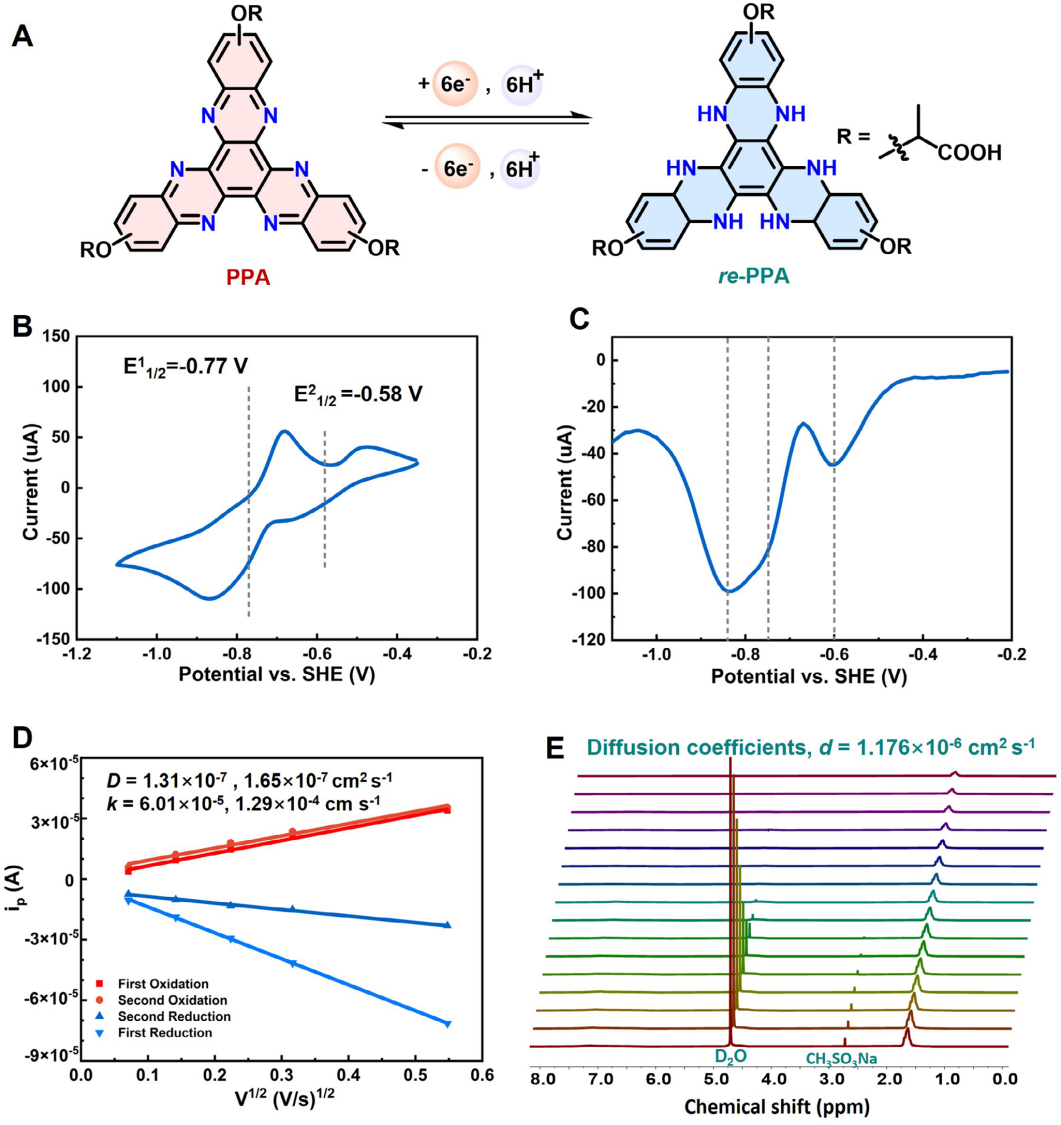
A) The redox reactions between **PPA** and ***re*‐PPA**. B) Cyclic voltammograms using a gold electrode for 25 mm **PPA** in 1.0 m KOH at the scan rate of 20 mV s^−1^, vs SHE. CV curves at different scan rates are available in Figure S8 (Supporting Information). C) Differential pulse voltammetry curves of 5 mm **PPA** in 1.0 m KOH. D) 5 mm **PPA** CV curves at different scan rates in 1.0 m KOH linearly fitted *i_p_* – *υ* ^1/2^ plot. E) Diffusion coefficients of **PPA** were determined via DOSY experiments (0.1 m **PPA** (K^+^)) in D_2_O containing 10 mm NaCH_3_SO_3_ as internal standard.

**PPA** is theoretically to have a six‐electron transfer process at full electrochemical capacity (**Figure** **2**A). To characterize its redox behavior, cyclic voltammetry (CV) studies were carried out first. CV analysis reveals that **PPA** exhibits two resolved redox couples at $E_{1/2}^{1}$= −0.77 V and $E_{1/2}^{2}$= −0.58 V vs SHE in 1.0 m KOH (Figure 2B). Differential pulse voltammetry (DPV) analysis of **PPA** revealed two well‐defined reduction peaks (Figure 2C), centered at −0.84 and −0.59 V, accompanied by a less distinct shoulder feature at −0.75 V, indicating the high electrochemical reversibility under pulsed conditions. While the CV curve for **PBA** shows unresolved redox couples, with the lowest redox wave estimated to be $E_{1/2}^{1}$= −0.68 V and $E_{1/2}^{2}$= −0.48 V vs SHE (Figure S7, Supporting Information). Considering redox reversibility and molecular solubility, **PPA** has been used in subsequent studies.

The diffusion coefficients (*D*) and the electron‐transfer rate constants (*k_0_*) of **PPA** were then evaluated using the Randles‐Sevcik method and Nicholson's treatment.^[^ ^35^, ^36^ ^]^ CV measurements of 5 mm **PPA** in 1.0 m KOH were conducted with varying scan rates ranging from 5 to 300 mV s^−1^. As CV analysis gave two well‐resolved redox waves, the measured diffusion coefficients of **PPA** were *D* = 1.31 × 10^−7^ cm^2^ s^−1^ and 1.65 × 10^−7^ cm^2^ s^−1^, for the respective redox waves. The corresponding electron‐transfer rate constants (*k_0_*) were *k_0_* = 6.01 × 10^−5^ cm s^−1^ and 1.29 × 10^−4^ cm s^−1^ for the first and the second redox couples (Figure 2D; Figures S8 and S9, Supporting Information). As CV measures the diffusion coefficient of redox‐active species at the electrode interface, diffusion‐ordered spectroscopy (DOSY) experiments with 0.1 m **PPA** in 1.0 m KOH were further conducted to investigate the molecular diffusion behavior in solution, independent of redox activity, giving an overall diffusion coefficient of *d* = 1.176 × 10^−6^ cm^2^ s^−1^ (0.01 m NaCH_3_SO_3_H as an internal standard) (Figure 2E). These combined studies reveal reversible redox behavior, fast kinetics, and multi‐electron transfer of **PPA** as a promising negative energy storage material (Table 1).

**Figure 3:**
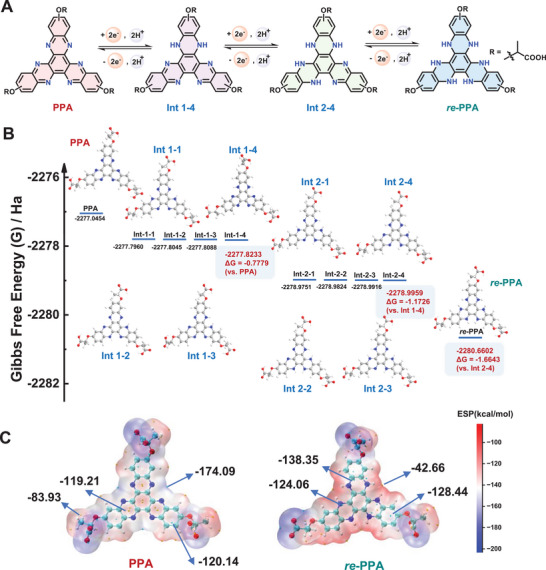
A) The proposed stepwise 2e^−^/2H^+^ PCET reactions between **PPA** and ***re*‐PPA**. B) Gibbs free energies of the species calculated in the charge‐discharge process. C) Calculated ESP distribution on the van der Waals surface of **PPA** and ***re*‐PPA**. Surface local minima and maxima of ESP are represented as green and orange spheres, respectively. The global maximum was labeled.

Apart from the redox reversibility of **PPA** characterized in electrochemical analyses, Pourbaix diagram analysis reveals a six‐electron redox mechanism for **PPA** (Figure S10, Supporting Information). The observed potential‐pH dependence exhibits a slope of −54 mV pH^−1^, closely approaching the theoretical −59 mV pH^−1^ expected for proton‐coupled electron transfer (PCET) processes involving one proton per electron. This correlation suggests a sequential three‐step redox mechanism, where each step comprises two‐electron and two‐proton transfer (2e^−^/2H^+^). DFT calculations were performed using a single isomer as the representative model for computational efficiency, which supports a stepwise PCET mechanism in **PPA**, governed by thermodynamic stabilization through selective electron‐proton localization (**Figure** **3**A; Table S2, Supporting Information). Initially, upon receiving 2e^−^/2H⁺, the **PPA** exhibits preferential localization of both electrons on a single phenazine ring with the lowest Gibbs free energy change (ΔG = −0.7779 Ha vs **PPA**) compared to other intermediates, forming **Int 1–4** (G = −2277.8233 Ha). Subsequent incorporation of an additional 2e^−^/2H⁺ pair drives the selective occupation of a second phenazine ring with the lowest Gibbs free energy change (ΔG = −1.1726 Ha vs **Int 1–4**), yielding the **Int 2–4** (G = −2278.9959 Ha). Finally, a third step 2e^−^/2H⁺ transfer completes the six‐electron reduction, giving the fully reduced product ***re*‐PPA** (G = −2280.6602 Ha, ΔG = −1.6643 Ha vs **Int 2–4)**. Consequently, as derived from electrochemical measurements and computational analysis, a three‐step, six‐electron redox mechanism of **PPA** in a KOH medium was proposed (Figure 3B).

Theoretical study on molecular electrostatic potential mapping (ESP) reveals distinct electrophilic and nucleophilic sites across its redox states. In the oxidized state **PPA**, the methyl group region exhibits a localized potential maximum of −83.93 kcal mol^−1^, while the area around the Nitrogen atom shows a localized potential minimum of −174.09 kcal mol^−1^. In ***re*‐PPA**, the potential maximum shifts to −42.66 kcal mol^−1^ around the Hydrogen atom of the N─H bonds, with a minimum of −138.35 kcal mol^−1^ on the phenazine skeleton (Figure 3C). The marked ESP shifts between **PPA** and ***re*‐PPA** drive charge delocalization across the molecular framework during electron transfer.

**Figure 4:**
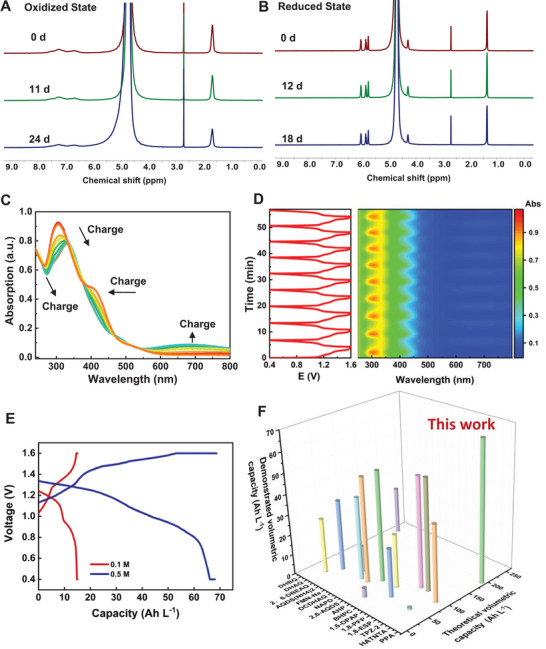
Time‐dependence ^1^H NMR spectra of A) **PPA** and B) ***re*‐PPA** (0.1 m in 1.0 m KOH at room temperature), diluted with D_2_O containing 10 mm NaCH_3_SO_3_ as the internal standard at a fixed volume ratio of 1:4. C) In situ UV–vis spectra of **PPA** during the charge–discharge process of one cycle. D) 2D mapping of in situ UV–vis spectra. E) The capacity vs voltage curves of 0.1 and 0.5 m **PPA** flow cells. F) A comparison of the theoretical and demonstrated volumetric capacity of various negolytes.

Time‐dependent ^1^H NMR analysis was performed to evaluate the chemical stability of **PPA** in both its oxidized and reduced states. Solutions of 0.1 m **PPA** and ***re*‐PPA** in 1.0 m KOH, with sodium methanesulfonate (NaCH_3_SO_3_) as an internal standard, were monitored over time at room temperature (**Figure** **4**A,B) and 45 °C elevated temperature (Figure S11, Supporting Information). They both exhibited superior stability, and no degradation products were detected over 18 days. Both in situ and ex situ UV–vis spectroscopic analysis were utilized to investigate the electrochemical behavior of **PPA** during charge–discharge cycling (Figure 4C; Figure S12, Supporting Information). The experiments were conducted under rigorously controlled conditions in an AORFB cell, employing a constant current density of 1.0 mA cm^−2^. The UV–vis spectra exhibited characteristic absorption bands for **PPA** in its oxidized state, with prominent peaks observed at ≈305 and 405 nm. Upon electrochemical reduction, two distinct new absorption features emerged at 335 and 680 nm, indicative of a significant alteration in the electronic configuration. This spectral evolution was fully reversible over successive charge‐discharge cycles. The reversible color change observed in the UV–vis spectra, as captured through 2D mapping during the charge and discharge cycles, illustrates the robust redox activity and stability of **PPA** (Figure 4D).

Moreover, in situ FTIR spectroscopy was used to tracke the molecular structure changes during cell cycling (Figure S13, Supporting Information). The transition from **PPA** to ***re*‐PPA** is characterized by the appearance of IR absorption bands at 1170 and 1450 cm^−1^, indicating the generation of a C─N single bond and a N─H single bond, respectively. This reversible transition is also accompanied by the periodic change of aromatic C═C stretching vibrations within the aromatic ring at 1507 and 1580 cm^−1^.

To further elucidate the redox mechanism, ex situ electron paramagnetic resonance (EPR) characterization was conducted on a 0.1 m **PPA** (K^+^) cell in 1.0 m KCl at 298 K across various states of charge (SOC) (Figure S14, Supporting Information). The absence of detectable EPR signals at different SOCs indicates that no radical species were generated during the charging process. This observation supports a stepwise two‐electron transfer process for **PPA**, where each redox step involves the transfer of an even number of electrons rather than single‐electron processes.

As **PPA** was expected to have a six‐electron transfer, the volumetric capacity of **PPA** was assessed using both 0.1 and 0.5 m solutions as the negolyte, paired with K_4_Fe(CN)_6_ as the posolyte. Galvanostatic‐potentiostatic charge–discharge profiles reveal the presence of two major voltage plateaus, which are consistent with the redox transitions identified in CV and DPV analysis. The 0.1 m cell delivers a volumetric capacity of 15.1 Ah L^−1^ with 94% capacity utilization (theoretically 16.1 Ah L^−1^ with six‐electron storage), while the 0.5 m cell achieves a capacity of 68.2 Ah L^−1^ with 85% capacity utilization (theoretically 80.4 Ah L^−1^ with six‐electron storage) (Figure 4E). The higher concentration cell exhibits an increased open circuit voltage (OCV) of 1.31 V at 50% SOC, attributed to concentration‐dependent pH shifts arising from PCET reactions, where pH variations modulate the Nernstian potential of the electrolytes. The demonstrated high‐concentration **PPA** cell with 3.0 m electron concentration gives a volumetric capacity of 68.2 Ah L^−1^. It outperforms other negolytes used in AORFBs, showing its great promise as a high‐capacity organic negolyte for energy storage systems (Figure 4F; Table S3, Supporting Information). Preparing for a long‐term cycling AORFB, the permeability of **PPA** was evaluated with an NC700 membrane. No detectable crossover was observed in 1.0 m KOH or 1.0 m KCl solution over more than one hundred days (Figure S15, Supporting Information). This exceptionally low permeability could be attributed to the large molecular structure of **PPA** and the electrostatic repulsion between the negatively charged carboxylate groups and the anionic surface of the NC700 membrane. These properties are beneficial for mitigating crossover‐induced capacity fade and performance losses in battery applications.

*The Application of PPA in AORFBs*. We initially assembled a 0.1 m **PPA** (in potassium salt, K^+^) cell in 7 mL 1.0 m KCl as negolyte, with a posolyte comprising 50 mL 0.15 m K_4_Fe(CN)_6_ and 0.01 m K_3_Fe(CN)_6_, separated by an NC700 as the cation‐exchange membrane. This neutral electrolyte was initially chosen as it offers a balance of high solubility and relatively low viscosity, facilitating good ionic mobility and stable electrochemical performance (Tables S4 and S5, Supporting Information). The **PPA**‐based AORFB with 0.6 m electron concentration was cycled at 20 mA cm^−2^ between 0.4 and 1.6 V, and the theoretical capacity was 16.1 Ah L^−1^. The cell accessed a capacity of 15.1 Ah L^−1^ with 94% capacity utilization, while the Coulombic efficiency always remained >99%. Following a cell cycling comprising 72 cycles of galvanostatic cycling, a further 399 cycles of galvanostatic‐potentiostatic cycling were performed. This galvanostatic‐potentiostatic cycling was employed to evaluate the long‐term battery stability, as it ensures complete utilization of the active material in each cycle by applying a constant‐voltage hold until the current drops to a predetermined cutoff. It provides a more accurate assessment of the molecular stability by effectively excluding contributions from incomplete charging or discharge.^[^ ^37^ ^]^

Under this estimation, the 0.1 m **PPA** (K^+^) cell demonstrated a low capacity fade rate of 0.006% per cycle, 0.07% per day, over a testing period exceeding 40.6 days (Figure S16, Supporting Information). Post‐cycling analysis of the electrolyte confirmed the absence of detectable degradation products, indicating its high electrochemical stability (Figure S17, Supporting Information). At 100% SOC of the 0.1 m **PPA** in 1.0 m KCl, the cell demonstrated a maximum power density of 0.146 W cm^−2^, while the OCV was 1.44 V. The polarization measurements revealed an area‐specific resistance (ASR) of 2.08 Ω cm^2^ for the cell at 50% SOC, and 2.26 Ω cm^2^ under fully charged conditions. The various current density behavior within a voltage cutoff of 0.4–1.6 V. At 10 mA cm^−2^, the cell delivered 85% of its theoretical six‐electron capacity. Nevertheless, Coulombic efficiencies >99% across all tested rates, with energy efficiency (EE) attaining 84% at 10 mA cm^−2^. The discharge capacity and EE decrease were observed with increasing current density, primarily ascribed to elevated ohmic polarization (Figure S18, Supporting Information).

The same concentration cell using 0.1 m **PPA** (K^+^) (0.6 m electron concentration) in 1.0 m KCl was constructed and operated at a higher current density of 40 mA cm^−2^ to access more cycling numbers of the battery for a further understanding of the molecular stability. When the current density was increased to 40 mA cm^−2^, the capacity utilization decreased to 61% and the EE dropped to 58%. This performance decrease can be attributed to increased mass transport limitations and higher polarization losses at elevated current densities, which reduce the EE. The cell underwent galvanostatic‐potentiostatic cycling for 2445 cycles, exhibiting exceptional stability with an ultralow capacity decay rate of 0.0017% per cycle, 0.05% per day, over a continuous testing for more than 80.5 days (Figure S19, Supporting Information). The long‐term cell cycling demonstrates exceptional electrochemical stability of **PPA** as a promising energy storage material.

To comprehensively evaluate **PPA**’s stability under alkaline conditions, a full cell employing 0.1 m **PPA** (K^+^) in 1.0 m KOH was assembled, with the negolyte comprised 7.0 mL of 0.1 m **PPA** (K^+^) paired with 50 mL of 0.15 m K_4_Fe(CN)_6_/0.01 m K_3_Fe(CN)_6_. The cell demonstrated 93% capacity utilization over a galvanostatic cycling for 45 cycles (3.7 days), followed by an extended galvanostatic‐potentiostatic 308 cycles (26.4 days). The system exhibited robust stability with a capacity decay rate of 0.007% per cycle (0.09% per day) (Figures S20 and S21, Supporting Information).

**Figure 5:**
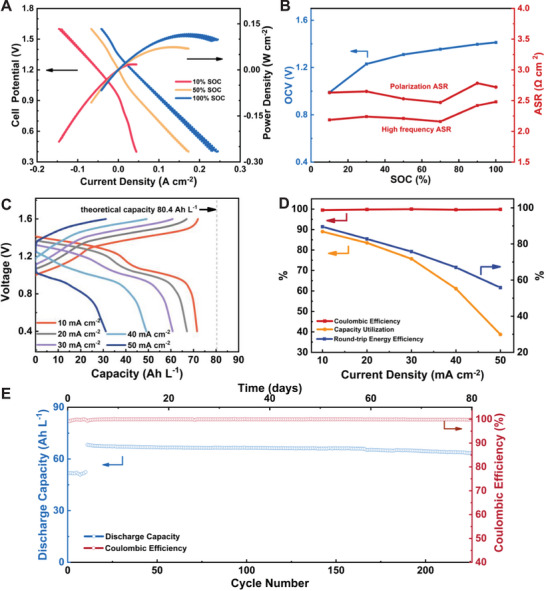
A) Cell potential and power density vs current density at 10%, 50%, and ≈100% SOC at room temperature of 0.5 m **PPA** (K^+^). B) Full‐cell OCV, high‐frequency, and polarization ASR vs various SOCs at room temperature of 0.5 m **PPA** (K^+^). C) Capacity and galvanostatic charge–discharge voltage profiles at various current densities; the vertical dashed line indicates the theoretical capacity. D) Capacity utilization, Coulombic efficiency, and round‐trip energy efficiency vs current density. E) Cycling performance of 0.5 m **PPA** (K^+^) in H_2_O. The cell was cycled galvanostatically at 20 mA cm^−2^ between 1.6 and 0.4 V, followed by a galvanostatic‐potentiostatic cycling for over 80 days.

A high‐concentration AORFB was constructed using 0.5 m **PPA** (K^+^) as negolyte, which was expected to deliver 3.0 m electron storage. Since the potassium form of **PPA** itself serves as an intrinsic ionic conductor, we selected pure water as the solvent without additional supporting electrolytes to minimize solution viscosity and reduce pumping energy losses (Figure S22 and Table S6, Supporting Information). Through systematic optimization to prevent water migration in this concentrated system, the final electrolyte configuration consisted of 5 mL 0.5 m **PPA** (K^+^) in water as the negolyte, paired with 20 mL 0.4 m K_4_Fe(CN)_6_, 0.6 m Na_4_Fe(CN)_6,_ and 0.4 m K_3_Fe(CN)_6_ in water as the posolyte. A charging current of 20 mA cm^−2^ was applied, and polarization curves were measured at 10, 50, and 100% SOC. At 100% SOC, the cell delivered with a peak power density of 0.117 W cm^−2^ (**Figure** **5**A). As the SOC increased from 10% to 100%, the OCV rose from 0.99 V at 10% SOC to 1.31 V at 50% SOC, reaching 1.41 V at 100% SOC (Figure 5B). High‐frequency EIS showed 2.48 Ω cm^2^ at 100% SOC, and the total impedance under DC polarization was 2.72 Ω cm^2^. The battery's rate capability of 0.5 m **PPA** (K^+^) in H_2_O was evaluated by applying current densities from 10 to 50 mA cm^−2^ in steps of 10 mA cm^−2^, under galvanostatic charge‐discharge cycling between 1.6 and 0.4 V (Figure 5C). Under these conditions, the Coulombic efficiency consistently exceeded 99.5%. At 10 mA cm^−2^, the cell achieved a capacity utilization of ≈89%, accompanied by a high round‐trip EE of 90% (Figure 5D). As the current density increased, the battery's capacity utilization and energy efficiency declined, primarily attributable to rising ohmic losses.

The cell was cycled galvanostatically at 20 mA cm^−2^ between 1.6 and 0.4 V for ≈10 cycles and 2.3 days. Then each galvanostatic half‐cycle was finished with a potential hold at the potential limit (1.6 V after charge, 0.4 V after discharge) until the magnitude of the current density fell below 4 mA cm^−2^. The battery performed galvanostatic‐potentiostatic cycling continuously for 77.7 days with a capacity of 68.2 Ah L^−1^ (85% capacity utilization) and a capacity decay rate of 0.032% per cycle, 0.095% per day over 80 days of continuous cycling (Figure 5E; Figure S22, Supporting Information), which outperforms other reported six‐electron systems (Table S4, Supporting Information). The viscosity of 0.1 m **PPA** (K^+^) in 1.0 m KCl is 1.23 × 10^−3^ Pa·s at room temperature, while 0.5 m **PPA** (K^+^) in water exhibits a forty times higher viscosity of 0.05 Pa·s (Table S6, Supporting Information). This obvious viscosity increase at elevated concentrations likely contributes to the reduced capacity utilization observed in the 0.5 m cell, posing limited mass transport within the electrolyte and increasing ohmic losses during charge–discharge cycles. Furthermore, post‐cycling analysis was conducted with a framework of CV, NMR, and UV–vis measurements (Figure S23, Supporting Information), which showed no detectable degradation and crossover of **PPA** after continuous cell charge–discharge cycles under the tested conditions. While our studies demonstrate the stability of **PPA** as a stable negative electrolyte, the possibility of slow, undetected degradation over extended year‐long operation cannot be fully excluded. Improvements in flow field design, membrane durability, and operational protocols could further increase the long‐term stability of the battery system.

## 3 Conclusion

In conclusion, we presented an ultra‐stable, high‐capacity AORFB based on a six‐electron storage molecule **PPA**, featuring a *π*‐extended fused‐ring conjugation system and branched carboxylate functional groups. The ether‐linked propanoic acid side chains disrupt *π*–*π* stacking while enhancing aqueous solubility to 7.2 m electron concentration (193.0 Ah L^−1^ theoretical capacity) and lowering the redox potential to −0.77 V vs SHE. Paired with a K_4_Fe(CN)_6_/K_3_Fe(CN)_6_ as posolyte, the **PPA**‐based AORFB achieves a cell voltage of 1.31 V at 3.0 m electron concentration, with an average Coulombic efficiency >99%. The system demonstrates exceptional cycling stability, with a capacity decay rate of 0.032% per cycle, 0.095% per day over 80 days of continuous operation. Long‐term battery cycling was also achieved with >99% Coulombic efficiency, low capacity decay of 0.0017% per cycle, 0.05% per day over 2445 cycles, 80.5 days. With a simple synthetic route starting from cost‐effective starting materials, **PPA** is demonstrated to be a scalable and promising energy storage material. This research provides an example of developing high volumetric capacity and long‐lifetime energy storage techniques for large‐scale energy storage.

## Conflict of Interest

A patent has been filed for this work.

## Supporting information



Supporting Information

## Data Availability

The data that support the findings of this study are available in the supplementary material of this article.
