# Supplementary material for: A Six‐Electron Energy Storage Material for Ultra‐Stable Aqueous Organic Redox Flow Batteries
Source: Adv Sci (Weinh). 2025 Sep 30;12(47):e14452. doi: 10.1002/advs.202514452 (PMC12713029; doi:10.1002/advs.202514452)
Supplement: Supplementary file 1 — Supporting Information [file ADVS-12-e14452-s001.pdf]

A Six-Electron Energy Storage Material for Ultra-Stable Aqueous Organic Redox Flow Batteries

Xiaowei Zhang[1,2], Lu Li,[2,3] Yunlong Ji[3], Pan Wang[1, 2, 4*]

[1] Department of Chemistry, Zhejiang University, Hangzhou, 310058, Zhejiang, China.

[2] Key Laboratory of Precise Synthesis of Functional Molecules of Zhejiang Province, Department of Chemistry, School of Science and Research Center for Industries of the Future, Westlake University, Hangzhou 310030, China.

[3] School of Chemistry and Materials Science, Hangzhou Institute for Advanced Study, University of Chinese Academy of Sciences, 1 Sub-lane Xiangshan, Hangzhou 310024, China.

[4] Division of Solar Energy Conversion and Catalysis at Westlake University, Zhejiang Baima Lake Laboratory Co., Ltd., Hangzhou 310000, Zhejiang, China.

* Corresponding author. E-mail: wangpan@westlake.edu.cn

Content

Supplementary Methods 2

Synthetic Procedures 3

Supplementary Note 1 - Solubility tests 8

Supplementary Note 2 - DOSY experiments 9

Supplementary Note 3 - Electrochemical characterization 10

Supplementary Note 4 - Permeability measurements 12

Supplementary Note 5 - Chemical stability experiments 13

Supplementary Note 6 - Theoretical studies 14

Supplementary references 43

Supplementary Methods

Unless stated otherwise, all air-sensitive reactions were carried out in oven-dried glassware by using standard Schlenk techniques. All solvents and reagents were obtained from commercial sources. ^{1}H NMR spectra were recorded on a Bruker Avance 500 MHz spectrometer. Chemical shifts were reported in ppm with the solvent resonance as the internal standard ($CHCl_3$, δ = 7.26 ppm or H_2O, δ = 4.79 ppm). Data for ^{1}H NMR were recorded as follows: chemical shift (δ, ppm), multiplicity (s = singlet, d = doublet, t = triplet, m = multiplet or unresolved, coupling constant (s) in Hz, integration). ^{13}C NMR (125 MHz) spectra were recorded on a Bruker Avance 500 MHz spectrometer. High-resolution mass spectra (HRMS) were obtained on the Waters Synapt-G2-Si Ultra-high Performance Liquid Chromatography-Time-of-Flight Mass Spectrometer using electrospray ionization (ESI). UV-vis spectra were recorded on Agilent Cary 60 spectrometer at room temperature. Viscosity was measured at room temperature with the Rheometer TA-Waters ARES-G2. Interfacial tensions were performed at room temperature with Dataphysics OCA 20 instrument using sessile drop method.

All flow cells were assembled with graphite runner for electrolyte flowing, cation-exchange membrane as separator, ELAT Hydrophilic carbon cloth (thickness 406 μm) as electrode (geometric surface area 5 cm^2), Viton (PVDF) gaskets for sealing the flow cell, and two copper current collectors, similar to our previous reports[1][2]. The flow of electrolytes was driven by a peristaltic pump. All electrochemical characterization was conducted and recorded on a BioLogic VSP-300 or BCS-128 instrument.

Synthetic Procedures

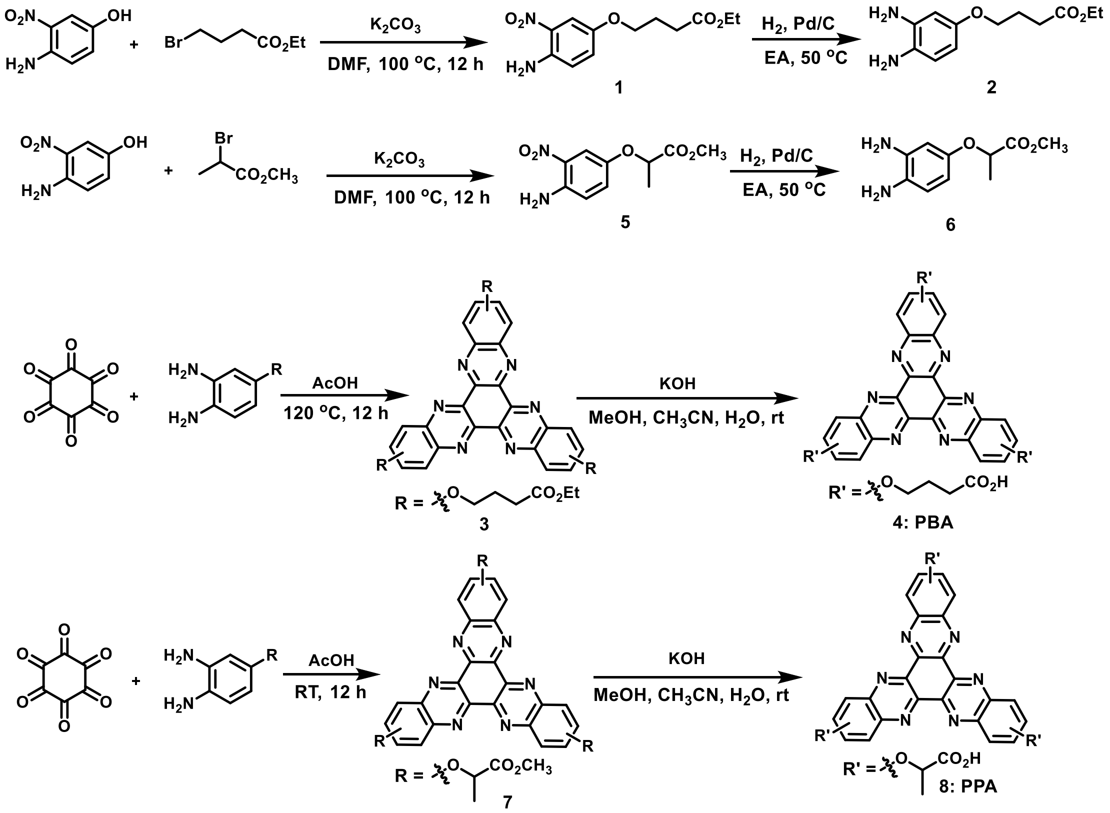

Scheme S1 Synthetic pathways of **PBA** and **PPA**.

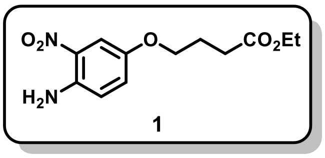

4-amino-3-nitrophenol (4.62 g, 30 mmol, 1.0 equiv.), ethyl 4-bromobutyrate (7.6 g, 39 mmol, 1.3 equiv.), K_2CO_3 (5.39 g, 39 mmol, 1.3 equiv.), and DMF (50 mL) were added to a 350 mL high-pressure flask, sealed with the N_2 atmosphere under the glove box, then moved from the glove box, heated and stirred at 100 °C overnight. After the reaction was completed, it was cooled to room temperature. Ethyl acetate (EA) and deionized water were added to the mixture, and the aqueous layer was extracted twice with EA. Then, Na_2SO_4 was added, and the organic phase was concentrated and purified via column chromatography with 10% - 20% EA in petroleum ether (PE) to collect product **1** as a deep red solid (6.87g, 86%). ^1H NMR (500 MHz, $CDCl_3$) δ 7.52 (d, J = 3.0 Hz, 1H), 7.04 (dd, J = 9.0, 2.5 Hz, 1H), 6.75 (d, J = 9.5 Hz, 1H), 5.90 (s, 2H), 4.14 (q, J = 14.5, 7.5 Hz, 2H), 3.97 (t, J = 6.0 Hz, 2H), 2.49 (t, J = 7.0, Hz, 2H), 2.12-2.07 (m, 2H), 1.26 (t, J = 7.0 Hz, 3H). ^{13}C NMR (125 MHz, $CDCl_3$) δ 173.1, 149.9, 140.0, 131.5,

127.0, 120.1, 107.3, 67.6, 60.5, 30.8, 24.5, 14.2 cm^{-1}. HRMS (ESI) m/z: $[M+H]^+$ calcd for $C_{12}H_{16}N_2O_5^+$ 269.1137, found: 269.1142.

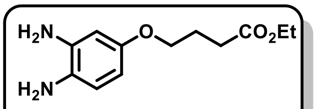

2

To a 100 mL PVDF flask that matched with the high-pressure hydrogenation reactor, the above-mentioned compound **1** (6.87 g, 25.6 mmol, 1 equiv.), Pd/C (10% on carbon, 687 mg), EA (40 mL) were added in air. The high-pressure hydrogenation reactor was sealed and filled with 40 bar hydrogen. The reaction mixture was vigorously stirred at 50 °C for 12 h under hydrogen atmosphere. After cooling to ambient temperature, excess hydrogen gas was carefully vented. The Pd/C catalyst was then removed by filtration through a sand funnel packed with diatomite. The filtrate was concentrated under reduced pressure to afford the crude product **2** as a purple oil (5.78 g, 95%). ^{1}H NMR (500 MHz, $CDCl_3$) δ 6.62 (d, *J* = 7.0 Hz, 1H), 6.31 (d, *J* = 2.0 Hz, 1H), 6.24 (dd, *J* = 7.0, 2.0 Hz, 1H), 4.14 (q, *J* = 12.0 , 6.0 Hz, 2H), 3.91 (t, *J* = 5.0 Hz, 2H), 3.20 (s, 4H), 2.48 (t, *J* = 6.0 Hz, 2H), 2.08-2.03 (m, 2H), 1.25 (t, *J* = 6.0 Hz, 3H). ^{13}C NMR (125 MHz, $CDCl_3$) δ 173.4, 153.8, 137.0, 127.4, 118.3, 105.0, 103.7, 77.3, 77.0, 76.8, 66.5, 60.4, 30.9, 24.8, 14.2 cm^{-1}. HRMS (ESI) m/z: $[M+H]^+$ calcd for $C_{12}H_{18}N_2O_3^+$ 239.1396, found: 239.1395.

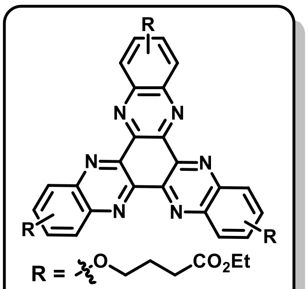

3

To a 350 mL high-pressure flask, cyclohexane-1,2,3,4,5,6-hexaone octahydrate (0.9 g, 2.6 mmol, 1.0 equiv.), **2** (2 g, 8.3 mmol, 3.2 equiv.) and AcOH (40 mL) were added, then the flask was sealed under N_2 atmosphere, and the mixture stirred at 120 ºC for 12 h. After completion of the reaction, cooled to room temperature, DCM and deionized water were added to the mixture, and the aqueous layer was extracted twice with DCM, then added the saturated $NaHCO_3$ solution to adjust the pH of the organic layers to neutral. The organic layers were then concentrated, and the crude product was purified by column chromatography using 25% - 50% EA in DCM to collect product **3** as a yellow solid (1.35 g, 68%). ^{1}H NMR (500 MHz, $CDCl_3$) δ 8.55-8.50 (m, 3H), 7.89-7.86 (m, 3H), 7.63 (tt, *J* = 10.0. 9.0 Hz, 3H), 4.31 (td, *J* = 6.0. 3.0 Hz, 6H), 4.19 (q, *J* = 14.0, 6.0 Hz,

6H), 2.62 (td, J = 7.0, 0.2 Hz, 6H), 2.32-2.27 (m, 6H), 1.29 (t, J = 7.0 Hz, 9H). ^{13}C NMR (125 MHz, $CDCl_3$) δ 172.9, 161.6, 145.3, 143.5, 140.3, 131.5, 126.6, 107.5, 67.9, 60.6, 30.9, 24.4, 14.3 cm^{-1}. HRMS (ESI) m/z: $[M+H]^+$ calcd for $C_{42}H_{43}N_6O_9^+$ 775.3091, found: 775.3091.

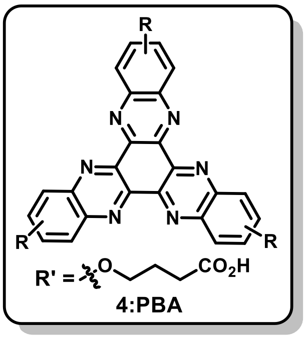

To a 250 mL round flask, **3** (1.35 g, 1.75 mmol, 1.0 equiv.), KOH (1.0 g, 17.46 mmol, 10 equiv.), THF (10 mL), CH_3CN (10 mL), H_2O (20 mL) were added, then the mixture stirred at room temperature for 10 h. After completion of the reaction, the mixture was concentrated under reduced pressure and dried in vacuo. The resulting residue was then acidified with 6.0 M HCl to adjust the pH to 1-2, which induced precipitation of a substantial amount of solid. The precipitate was collected via vacuum filtration, copiously washed with deionized water, and subsequently dried in vacuo at 50°C overnight, affording the desired product **PBA** as a yellow solid (0.96 g, 81%). ^1H NMR (500 MHz, DMSO-d_6) δ 12.26 (s, 3H), 8.14-8.03 (m, 3H), 7.57-7.46 (m, 3H), 7.35 (d, J = 101.5 Hz, 3H) 4.28-4.17 (m, 6H), 2.55 (t, J = 7.0 Hz, 6H), 2.15-2.11 (m, 6H). ^{13}C NMR (125 MHz, DMSO-d_6) δ 174.6, 160.5, 143.7, 142.2, 140.1, 138.5, 130.6, 125.4, 106.7, 67.9, 30.6, 24.5 cm^{-1}. HRMS (ESI) m/z: $[M+H]^+$ calcd for $C_{36}H_{31}N_6O_9^+$ 691.2153, found: 691.2158.

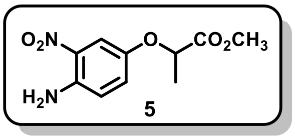

4-amino-3-nitrophenol (9.24 g, 60 mmol, 1.0 equiv.), 2-bromopropanoate (13.1 g, 78 mmol, 1.3 equiv.), K_2CO_3 (10.76 g, 78 mmol, 1.3 equiv.), and DMF (100 mL) were added to a 350 mL high-pressure flask, sealed with the N_2 atmosphere under the glove box, then moved from the glove box, heated and stirred at 100 °C overnight. Upon completion of the reaction, the mixture was cooled to room temperature and partitioned between EA and deionized water. The aqueous layer was further extracted with EA (2 × 30 mL). The combined organic phases were dried over Na_2SO_4, filtered, and concentrated under reduced pressure. The crude residue was subsequently purified by silica gel column chromatography using 10% - 20% EA in PE to collect product **5** as orange solid (12.32 g, 87 %). ^1H NMR (500 MHz, $CDCl_3$) δ 7.52 (d, J = 3.0 Hz, 1H),

7.12 (dd, J = 9.6, 3.0 Hz, 1H), 6.76 (d, J = 9.6 Hz, 1H), 5.90 (s, 2H), 4.72 (q, J = 13.2, 6.6 Hz, 1H), 3.77 (s, 3H), 1.61 (d, J = 6.6 Hz, 3H). ^{13}C NMR (125 MHz, $CDCl_3$) δ 172.2, 148.5, 140.5, 131.4, 127.5, 120.2, 109.2, 73.5, 52.4, 18.4 cm^{-1}. HRMS (ESI) m/z: $[M+H]^+$ calcd for $C_{10}H_{12}N_2O_5^+$ 241.0824, found: 241.0820.

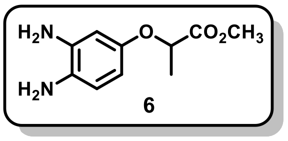

To a 100 mL PVDF flask that matched with the high-pressure hydrogenation reactor, the above-mentioned crude products **5** (12.3 g, 52.2 mmol, 1.0 equiv.), Pd/C (10% on carbon, 1.2 g), EA (60 mL) were added in air. The high-pressure hydrogenation reactor was sealed and filled with 40 bar hydrogen. The reaction mixture was vigorously stirred at 50°C for 12 h under hydrogen atmosphere. After cooling to ambient temperature, excess hydrogen gas was carefully vented. The Pd/C catalyst was then removed by filtration through a sand funnel packed with diatomite. The filtrate was concentrated under reduced pressure to afford the crude product **6** as a black oil (10.0 g, 93% yield). ^{1}H NMR (500 MHz, $CDCl_3$) δ 6.58 (d, J = 8.5 Hz, 1H), 6.33 (d, J = 2.5 Hz, 1H), 6.20 (dd, J = 8.5 Hz, 3.0 Hz, 1H), 4.63 (q, J = 14.0, 7.0 Hz, 1H), 3.74 (s, 3H), 1.56 (d, J = 7.0 Hz, 3H). ^{13}C NMR (125 MHz, $CDCl_3$) δ 173.2, 152.3, 137.0, 128.4, 118.0, 105.7, 104.7, 73.4, 52.2, 18.7 cm^{-1}. HRMS (ESI) m/z: $[M+H]^+$ calcd for $C_{10}H_{14}N_2O_3^+$ 211.1083, found: 211.1077.

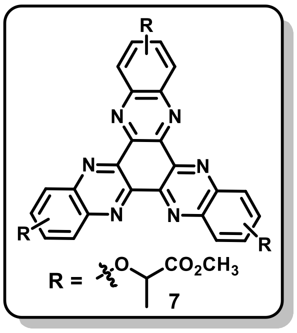

To a 350 mL high-pressure flask, cyclohexane-1,2,3,4,5,6-hexaone octahydrate (3.9 g, 12.5 mmol, 1.0 equiv.), **6** (8.4 g, 40 mmol, 3.2 equiv.) and AcOH (100 mL) were added, then the flask was sealed under N_2 atmosphere, and the mixture was stirred at room temperature for 24 h. Upon completion of the reaction, the mixture was partitioned between DCM and deionized water. The aqueous layer was further extracted with DCM, and the combined organic phases were washed with saturated $NaHCO_3$ to adjust the pH to neutral. After drying over Na_2SO_4, the solution was filtered and concentrated under reduced pressure to afford the crude product and purified via column chromatography using 25% - 50% EA in DCM to collect product **7** as a yellow solid (7.85 g, 91% yield). ^{1}H NMR (500 MHz, $CDCl_3$) δ 8.58-8.54 (m, 3H), 7.76-7.71 (m, 6H), 5.12-5.08 (m, 3H), 3.87 (s, 9H),

1.79 (d, J = 7.0 Hz, 9H). ^{13}C NMR (125 MHz, $CDCl_3$) δ 171.4, 160.4, 145.0, 143.4, 141.4, 140.3, 131.8, 126.9, 107.9, 72.9, 52.7, 18.6 cm^{-1}. HRMS (ESI) m/z: $[M+H]^+$ calcd for $C_{36}H_{30}N_6O_9^+$ 691.2153, found: 691.2149.

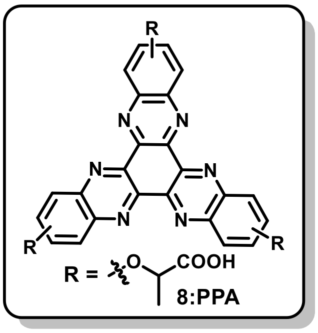

To a 250 mL round flask, **7** (7.85 g, 11.38 mmol, 1.0 equiv.), KOH (7.0 g, 113 mmol, 10 equiv.), and H_2O (50 mL) were added, then the mixture was stirred at room temperature for 8 h. After completion of the reaction, the mixture was then concentrated and further dried under vacuum. 6.0 M HCl was used to adjust the pH to 1-2, and a large amount of solid was precipitated. The precipitations were collected by filtration, washed with water and further dried under vacuum to collect product **PPA** as a yellow solid (6.35 g, 86% yield). ^{1}H NMR (500 MHz, DMSO-d_6) δ 13.41 (s, 3H), 8.21-8.10 (m, 3H), 7.66-7.37 (m, 6H), 5.30-5.20 (m, 3H), 1.75-1.72 (m, 9H). ^{13}C NMR (125 MHz, DMSO-d_6) δ 173.0, 159.9, 143.8, 140.2, 138.8, 131.2, 126.0, 107.8, 72.9, 18.7 cm^{-1}.

^{1}H NMR (500 MHz, D_2O) δ 7.81 (3H), 7.40 (3H), 6.80 (3H), 1.79 (9H). ^{13}C NMR (125 MHz, D_2O) δ 178.9, 160.0, 143.1, 139.4, 137.6, 129.7, 126.4, 105.7, 75.3, 18.3 cm^{-1}. HRMS (ESI) m/z: $[M+H]^+$ calcd for $C_{33}H_{24}N_6O_9^+$ 649.1683, found: 649.1671.

Supplementary Note 1 - Solubility tests

The solubility of **PBA** or **PPA** was measured by adding the **PBA** or **PPA** (K^+) compounds to the corresponding electrolyte solutions until no further solids could be dissolved. The supernatant was taken out after centrifugation to obtain the saturated solution of **PBA** or **PPA** (K^+). The saturated solution was then diluted by a known amount and the concentration was determined by UV-vis spectrophotometry (Agilent Cary 60 spectrometer). Following centrifugation at 8,000 rpm for 15 min, the supernatant containing the saturated **PBA/PPA** (K^+) solution was carefully decanted. This saturated solution was subsequently diluted with corresponding electrolyte solutions. The concentration was calculated according to a pre-calibrated absorbance versus concentration curve of known concentrations of **PBA** or **PPA** (K^+).

Supplementary Note 2 - DOSY experiments

DOSY experiments were performed at 25 °C on a Bruker NEO 600 MHz NMR spectrometer (600.23 MHz for proton frequency) equipped with a QCI-F Cryoprobe. For 1D ^{1}H experiments, 64 k complex data points were acquired with 16 scans. DOSY experiments used the "ledbpgp2s" pulse sequence to measure the self-diffusion coefficient D, with a relaxation delay of 3.0 s and 16 scans in total.16 linear steps from 2% to 95% of gradient strength, and a t2 (F2 dimension) of 16k sampling data points were used. The implemented diffusion time, big delta, and the diffusion gradient length, little delta, were 60 and 1.5 ms, respectively. Processing was carried out using Topspin 4.0.7. To obtain the relationship between molecular weight (MW) and self-diffusion coefficients, the method published by Gareth A. Morris was employed.[3] Diffusion values were obtained with an exponential model in Bruker TopSpin software.

Pulsed field gradient NMR spectroscopy, also known as q-space imaging, enables the measurement of molecular translational diffusion. By applying a magnetic field gradient, molecules are spatially encoded according to their position within the sample tube. If diffusion occurs during the interval Δ after this initial encoding, a second gradient is used to decode their new positions. The resulting NMR signal, integrated over the entire sample volume, exhibits attenuation that depends on both the diffusion time Δ and the gradient properties (g, δ). This attenuation behavior is mathematically described by

$$I = I_0\, e^{-D\gamma^2 g^2 \delta^2 (\Delta - \frac{\delta}{3})}$$

Here, I represents the observed signal intensity, I_0 is the reference intensity (i.e., the signal without attenuation), D denotes the diffusion coefficient, γ is the gyromagnetic ratio of the nucleus under observation, g stands for the gradient strength, δ is the duration of the gradient pulse, and Δ is the diffusion time. For simplicity, these parameters are often grouped together in practice.

Supplementary Note 3 - Electrochemical characterization

(1) The cyclic voltammetry (CV) measurements for **PPA** were conducted in a standard three-electrode configuration comprising: (i) a gold disk working electrode (diameter = 2 mm,), (ii) an Ag/AgCl reference electrode (3.0 M KCl salt bridge solution), and (iii) a platinum wire counter electrode. CV was conducted with 25.0 mM **PPA** (K^+) in 1.0 M KOH while the CV was tested at a sweep rate of 20 mV s^{-1}.

(2) The CV measurements for **PBA** were conducted in a standard three-electrode configuration comprising: (i) a carbon cloth working electrode (area = 0.5 cm×1.0 cm), (ii) an Ag/AgCl reference electrode (3.0 M KCl salt bridge solution), and (iii) a platinum wire counter electrode. CV was conducted with 25.0 mM **PBA** (K^+) in 2.0 M KOH while the CV was tested at a sweep rate of 20 mV s^{-1}.

(2) The diffusion coefficients (D) and charge transfer rate constants (k_0) were calculated according to an established literature method [4]. For electrochemically reversible systems, D was typically evaluated using the Randles-Sevcik equation (Eq. 1). The criterion for reversibility was defined as a peak separation $\Delta E_p \leq$ (57/n) mV [5].

$$i_p = 0.4463nFAC(\frac{nFvD}{RT})^{1/2} \quad (1)$$

where i_p is the peak current, n represents the number of electrons transferred, F is the Faraday constant (96485 C mol^{-1}), A refers to the electrode surface area (A = 0.034 cm^2), C is the bulk concentration of **PPA** (5 mM), v is the scan rate and T is the room temperature (298 K). The diffusion coefficient D is derived from the slope of the linearly fitted $i_p - v^{1/2}$ plot, based on the relationship $2.69\times10^5\ n^{3/2}ACD^{1/2}$. In the case of an irreversible redox reaction (identified by $\Delta E_p >$ (200/n) mV, a correction factor α was introduced, leading to the Eq. 2:

$$i_p = 0.4463nFAC(\frac{n\alpha FvD}{RT})^{1/2} \quad (2)$$

Here, α is defined by Eq. 3:

$$|\boldsymbol{E_p} - \boldsymbol{E_{p/2}}| = (\frac{48}{an})\ \boldsymbol{mV} \quad (3)$$

where E_p and $E_{p/2}$ represent the potentials at the peak current and at half of the peak current, respectively. Using Eq. 2, the D for the anodic and cathodic peaks (D_O and D_R)

were determined. The overall diffusion coefficient D was taken as the average of value of D_O and D_R. Furthermore, based on the CV curves obtained at various scan rates, the linear relationship of $ln(i_p)$ and the overpotential (E_p -$E^{0'}$), as described by in Eq. 4, was utilized to estimate the k_0:

$$ln(i_p) = ln(0.227nFAk^0C^*) - (\alpha F/RT)(E_P - E^{0'}) \qquad (4)$$

Here, C^* is the bulk concentration of **PPA** (5 mM), $E^{0'}$ is the formal potential and is determined from Eq. 5:

$$E^{0'} = \frac{\sum_{i=1}^{j} \frac{(E_{pai}+E_{pci})}{2}}{j} \qquad (5)$$

where j denotes the total number of CV scans, while E_{pa} and E_{pc} correspond to the anodic and cathodic peak potentials, respectively. The linearly fitted plot of $ln(i_p)$ vs. (E_p − $E^{0'}$) yields an intercept that is functionally related to the charge transfer rate constant k_0, as shown in Figure S9.

(3) Polarization measurements and cell cycling. (a) The 0.1 M **PPA** cell of 1.0 M KCl or 1.0 M KOH as supporting electrode was assembled with 0.1 M **PPA** (K^+) in 1.0 M KCl or 1.0 M KOH as negolyte (7.0 mL), 0.15 M $K_4Fe(CN)_6$ and 0.01 M $K_3Fe(CN)_6$ in 1.0 M KCl or 1.0 M KOH as posolyte (50 mL), with NC700 as exchange membrane. Each side contained three carbon cloths of AvCarb Felt G100 as the electrode. (b) The high concentration cell was assembled with 0.5 M **PPA** (K^+) in H_2O as negolyte (5.0 mL), 0.4 M $K_4Fe(CN)_6$, 0.6 M $Na_4Fe(CN)_6$, and 0.4 M $K_3Fe(CN)_6$ in H_2O as posolyte (20 mL), with NC700 as exchange membrane. Each side contained two carbon cloths of AvCarb Felt G100 as the electrode. The test temperature was held at room temperature.

(4) *In situ* UV-vis measurement. The posolyte comprised 10 mL of a mixed solution containing 10 mM $K_4Fe(CN)_6$ and 0.1 mM $K_3Fe(CN)_6$ dissolved in 1.0 M KOH electrolyte. The negolyte consisted of 15 mL of 1.85×10^{-2} mM **PPA** solution in 1.0 M KOH with NC700 as the exchange membrane. The test was conducted at room temperature, with each side containing two carbon cloths of AvCarb Felt G100 as the electrode.

(5) *In situ* FTIR measurement was performed using a Mettler-Toledo ReactIR-15

instrument. The posolyte comprised 20 mL of a mixed solution containing 0.2 M $K_4Fe(CN)_6$, 0.3 M $Na_4Fe(CN)_6$, and 0.2 M $K_3Fe(CN)_6$ dissolved in 1.0 M KCl electrolyte. The negolyte consisted of 5 mL of 0.3 M **PPA** (K^+) solution in 1.0 M KCl with NC700 as exchange membrane. The test was conducted at room temperature, with each side containing two carbon cloths of AvCarb Felt G100 as the electrode.

Supplementary Note 4 - Permeability measurements

The permeability of the **PPA** (K^+) across the NC700 membrane was evaluated with a commercially customer-made electrolyzer with a two-compartment cell according to the previously published[1]. The donating side was filled with the 0.1 M **PPA** (K^+) in 1.0 M KOH or 1.0 M KCl while the receiving side was filled with 1.0 M KOH or 1.0 M KCl in the same volume, both sides of the cell were continuously stirred. The solution was taken out from the receiving side at different time intervals for UV-vis spectrophotometry and then put back to the receiving side after characterization. The concentration was calculated from a calibration curve of UV-vis absorption at different concentrations and the permeability of **PPA** (K^+) was calculated[2][3]based on Fick's law as the equation: $P = \frac{\Delta \ln (1-\frac{2C_t}{C_0})(\frac{V_0 l}{2A})}{\Delta t}$ where P is permeability (cm^2 s^{-1}), A is the effective membrane area (cm^2), t is elapsed time (s), C_t (mol L^{-1}) is the concentration of **PPA** (K^+) which has crossed the membrane from the donating side and is detected by the UV-vis at time, V_0 is the volume of the solution in either compartment (5 cm^3), I is the thickness of the NC700 membrane (15 μm), C_0 is the concentration of **PPA** (K^+) in the donating side at time zero (0.1 mol L^{-1}), and Δ represents a finite difference.

Supplementary Note 5 - Chemical stability experiments

Samples of the oxidized state were prepared in advance, and the ^{1}H NMR spectra was recorded at intervals. Samples of the reduced state were prepared by fully charging in cells, stored in a glove box, and recorded at intervals for ^{1}H NMR spectra. All samples were stored at the same temperature as the cycling condition (room temperature / 45 °C). In the cycling stability experiments, 0.1 mL of cycling sample was taken from the negolyte before and after the cycling for ^{1}H NMR spectra, respectively. All samples were diluted with D_2O containing the internal standard (10 mM $NaCH_3SO_3$) at a fixed volume ratio of 1:4.

Supplementary Note 6 - Theoretical studies

All density functional theory[6] (DFT) calculations were performed with the Gaussian 16. The geometry optimizations and frequency calculations were performed using B3LYP functional with 6-31+G(d,p)[7] basis set, while single point calculations were performed using B3LYP/6-311+G(d,p)[8]. The D3 dispersion correction[9] was added to ensure a proper description of dispersive interactions. The implicit SMD solvent mode[10] was used to represent the solvent effect of the water molecule. The solubility was calculated by the difference between the single point energy of the gas phase and solution phase at M05-2X/6-31G*[11] level of theory.

The detail of Simulation details and methodology: To reduce the dependence on molecular conformation in molecular modeling, the GAFF2 force field[12] parameters are obtained using acpype code[13], and RESP atomic charges under B3LYP-D3(BJ)/6-31G(d,p) level[14] are calculated by Gaussian 16 A.03 software [15]. Molecular dynamics simulations are performed using GROMACS 2021.7 software[16]. Long-range electrostatic interactions are treated with the particle mesh Ewald (PME) method[17] with 1.2 nm as the Coulomb cutoff, and the van der Waals (vdW) interactions are treated with the force-switching method[18], where the forces smoothly decayed to zero between 0.9 and 1.2 nm to reduce the cutoff noise. The LINCS algorithm[19] is used to apply the bond constraint related to hydrogen, and a dispersion correction is used for both energy and pressure. All the simulation systems are energy-minimized by 10000 steps steepest descent with a time step of 1 fs, and then undergo 1 ns of NPT pre-equilibration run using leap-frog MD integrator[20] before launching a long time (100 ns) production run with a time step of 2 fs. During all the simulations, temperature is kept constant at 298.15K by using the velocity rescaling (V-rescale) thermostat[21] (with τ_t = 2.0 ps). The initial size of the simulated box is 6 nm×6 nm×6 nm and a three-dimensional periodic boundary condition (PBC) is used. The IGM model[22] was used to visualize the intermolecular interactions by Multiwfn 3.8 (dev) software[23]. Results are visualized using VMD 1.9.3[24] software.

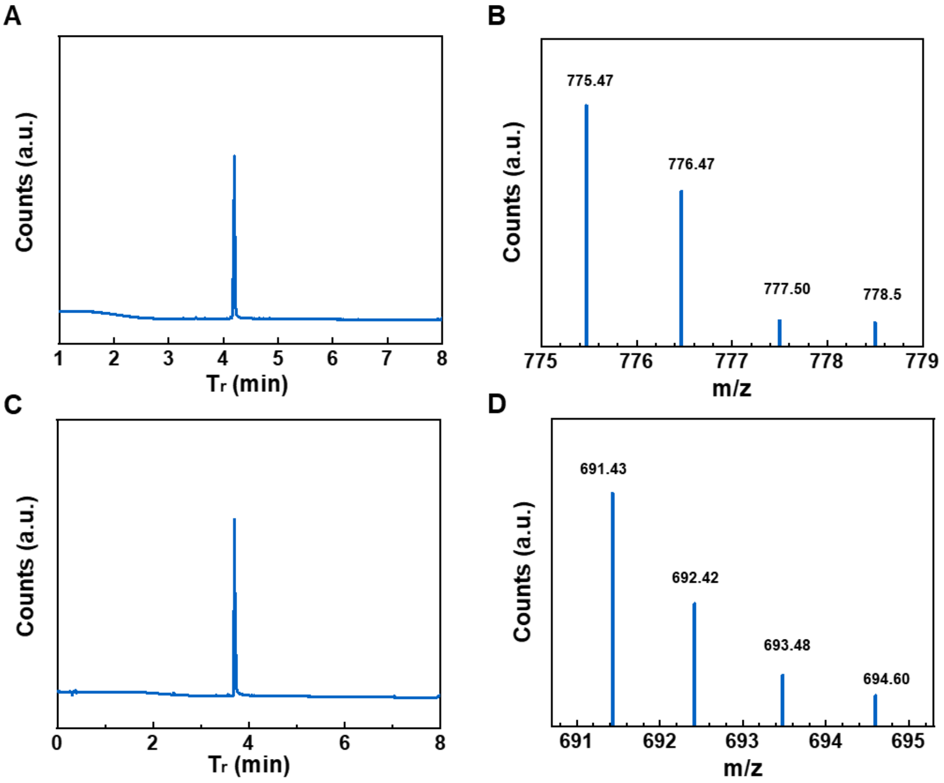

Figure S1 (A) The liquid chromatography trace of **PBA**. (B) The selected mass spectrum of **PBA**. (C) The liquid chromatography trace of **PPA**. (D) The selected mass spectrum of **PPA**.

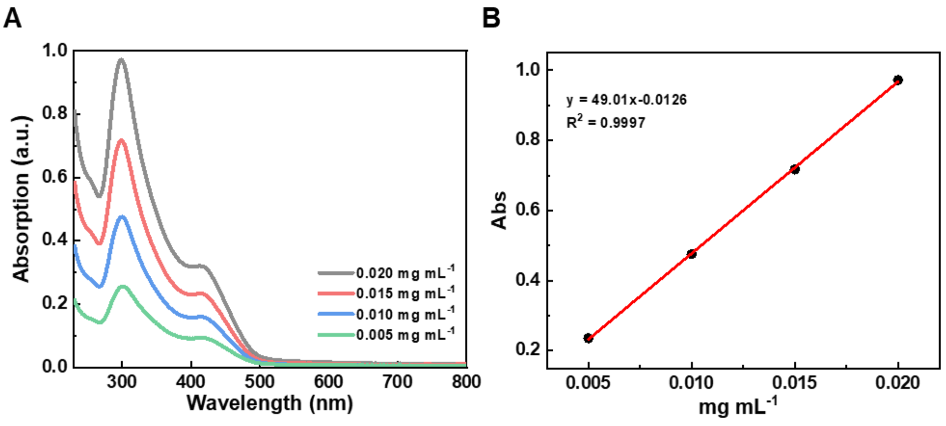

Figure S2 (A) UV-vis absorbance versus wavelength at various concentrations in 1.0 M KOH solution. (B) The fitted calibration curve of several known concentrations of **PBA**.

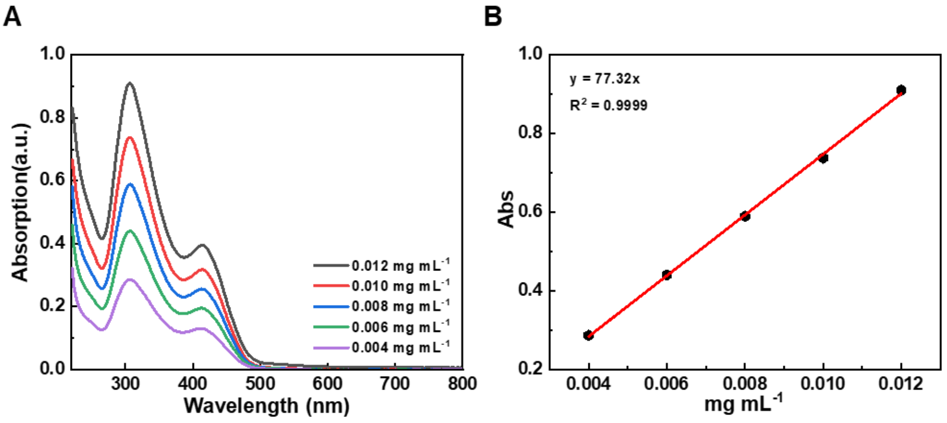

Figure S3 (A) UV-vis absorbance versus wavelength at various concentrations in 1.0 M KOH solution. (B) The fitted calibration curve of several known concentrations of **PPA**.

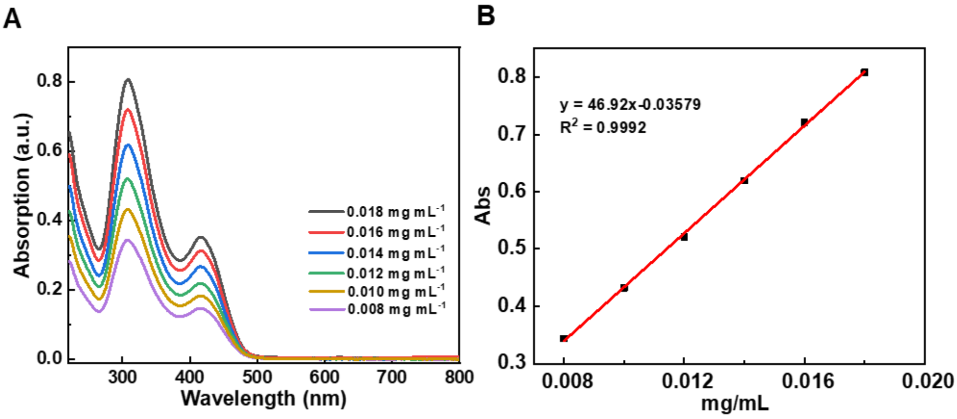

Figure S4 (A) UV-vis absorbance versus wavelength at various concentrations in 1.0 M KCl solution. (B) The fitted calibration curve of several known concentrations of **PPA**.

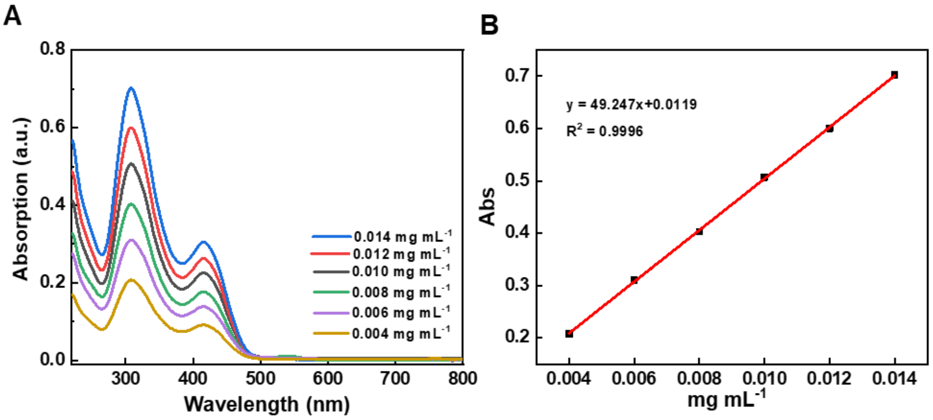

Figure S5 (A) UV-vis absorbance versus wavelength at various concentrations in H_2O. (B) The fitted calibration curve of several known concentrations of **PPA**.

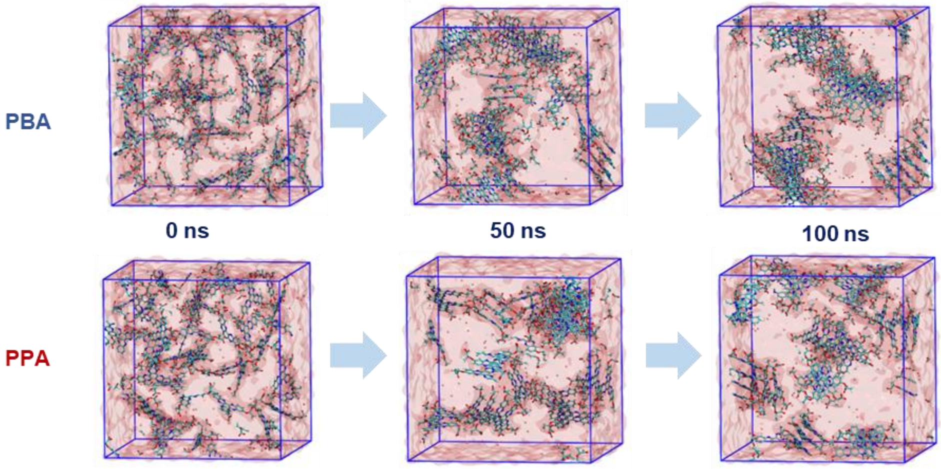

Figure S6 Molecular dynamics simulations snapshots of 300 mM **PBA** and **PPA** in H_2O. Snapshots of the front view of **PBA** and **PPA** for 0 ns, 50 ns, and 100 ns which represent contained water molecules.

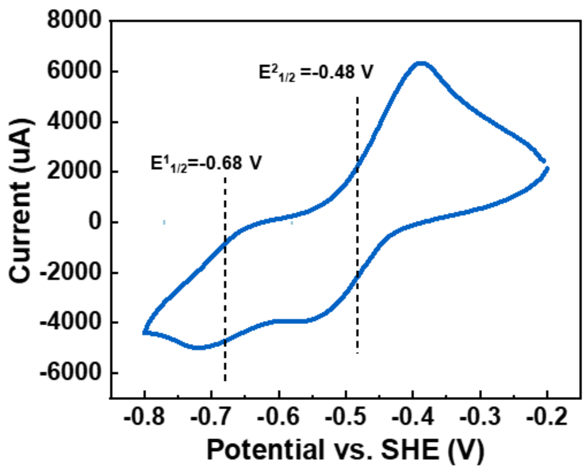

Figure S7 CV measurements of **PBA** (K^+) in 2.0 M KOH solution.

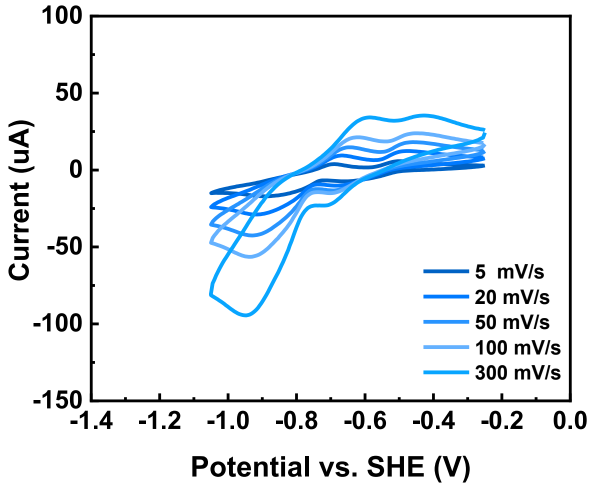

Figure S8 CV curves at different scan rates (5 mM **PPA**, 1.0 M KOH).

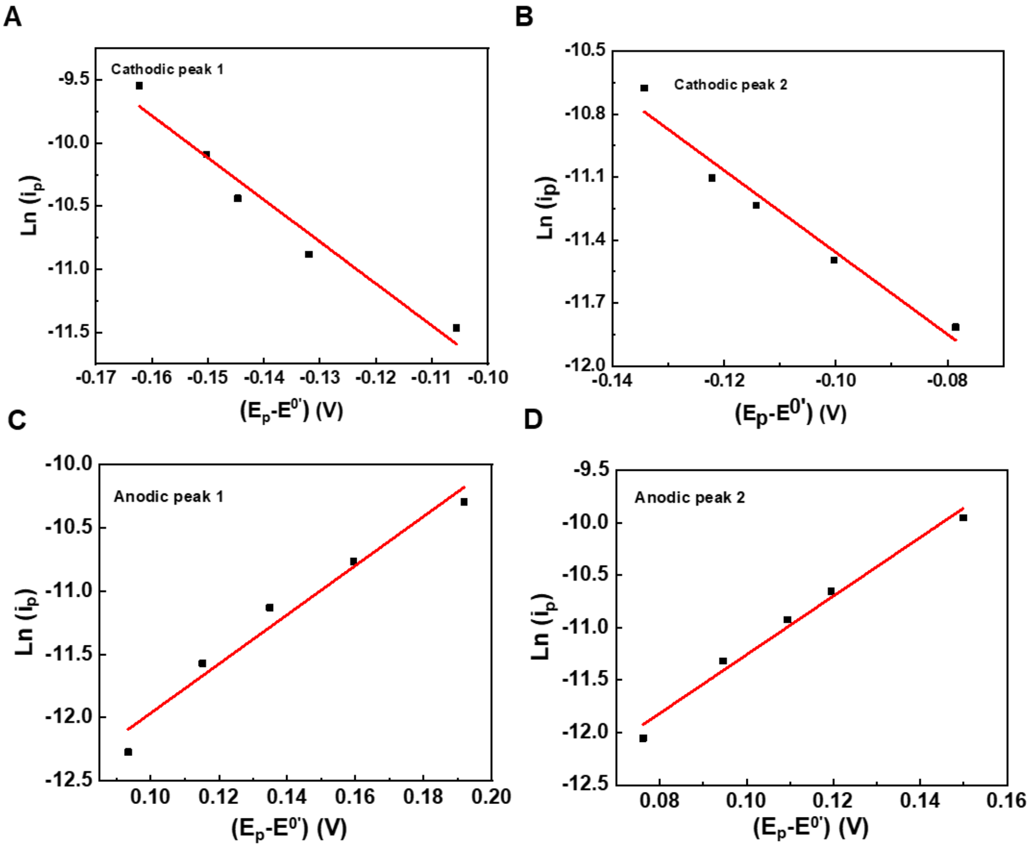

Figure S9 (A) Linearly fitted $ln(i_p) – (E_p\text{-}E^{0'})$ plot for the first cathodic peak of 5 mM **PPA**. (B) Linearly fitted $ln(i_p) – (E_p\text{-}E^{0'})$ plot for the second cathodic peak of 5 mM **PPA**. (C) Linearly fitted $ln(i_p) – (E_p\text{-}E^{0'})$ plot for the first anodic peak of 5 mM **PPA**. (D) Linearly fitted $\ln(i_p) –$ $(E_p\text{-}E^{0'})$ plot for the second anodic peaks of 5 mM **PPA**.

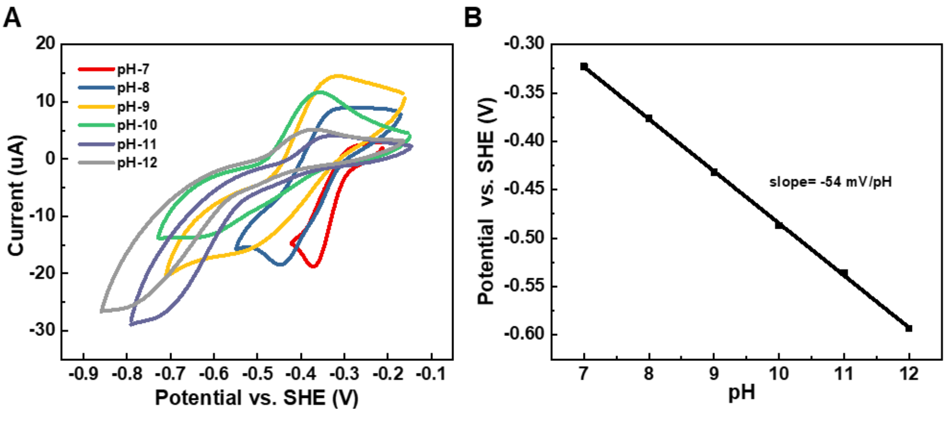

Figure S10 (A) CV curves of **PPA** at varying pH from 7.0 to 12.0. (B) Pourbaix diagram. The y-axis potential refers to the $E_{1/2}$ of the redox couples. Test temperature: 298 K.

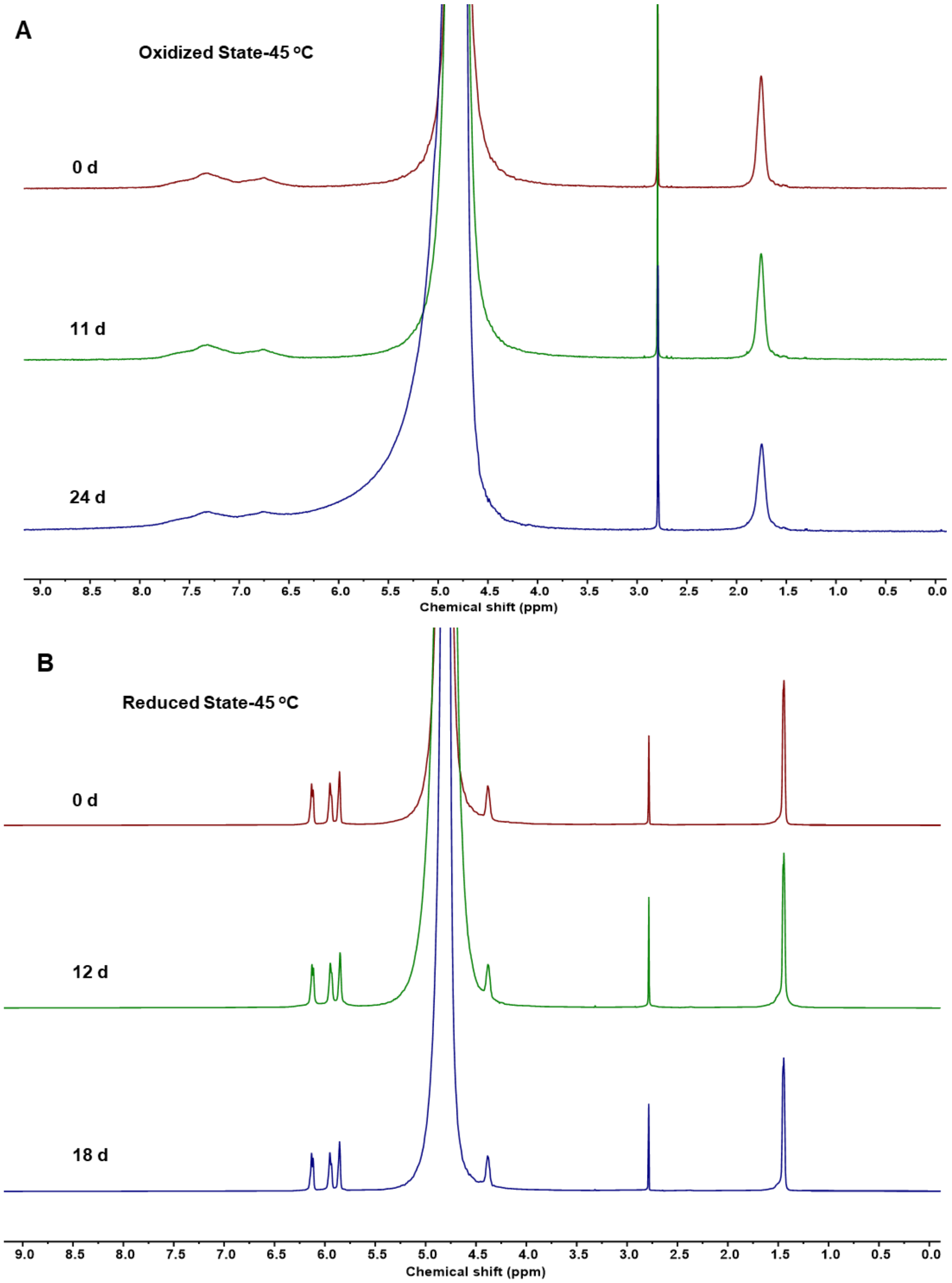

Figure S11 Time-dependence ^{1}H NMR spectra of (A) **PPA** (K^+) and (B) ***re*-PPA** (K^+) (0.1 M in 1.0 M KOH negolyte solution at 45 ºC).

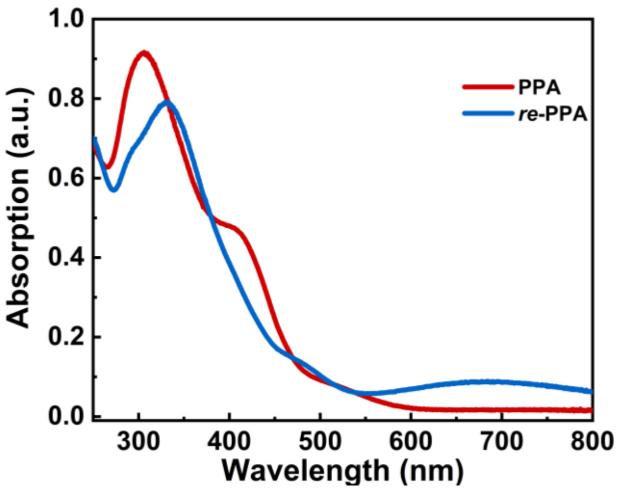

Figure S12 *Ex situ* UV-vis spectra of **PPA** and ***re*-PPA**.

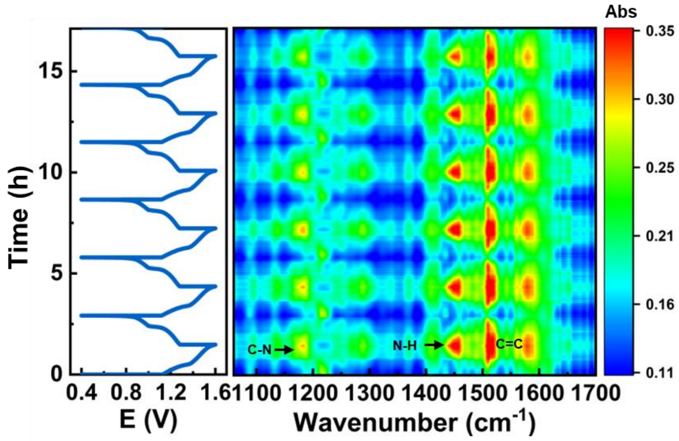

Figure S13 *In situ* FTIR spectra of **PPA** (K^+) at 298 K in 1.0 M KCl.

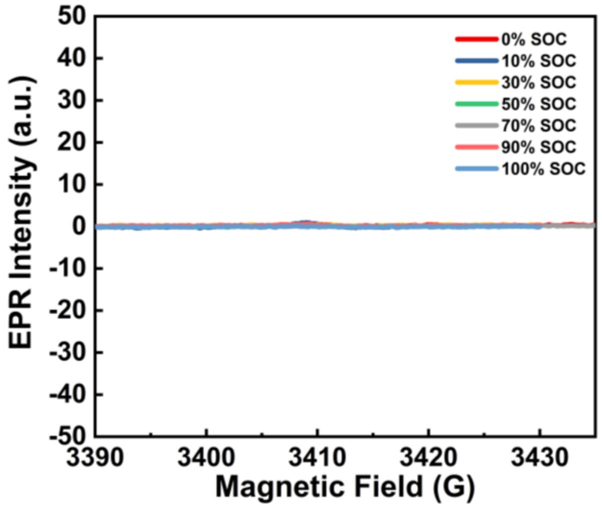

Figure S14 EPR spectra of **PPA** (K^+) at 298 K with 0.1 M concentration in 1.0 M KCl. EPR measurements were performed on a continuous wave X-band electron paramagnetic resonance (CW EPR) spectrometer from CIQTEK. Experimental parameters for solution-based EPR measurements are provided as follows: modulation amplitude = 1.0 G, attenuation = 30 dB, receiver harmonic = 1, microwave power = 0.1 mW, and frequency = 9.56 GHz.

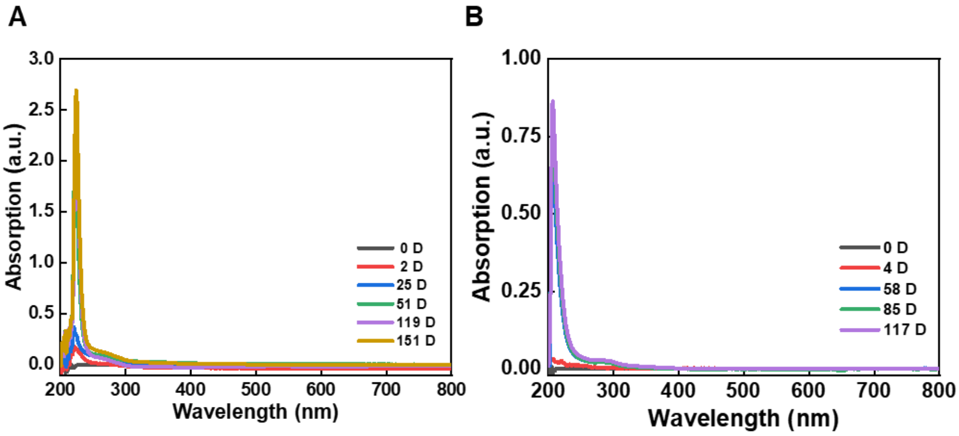

Figure S15 The permeability of the **PPA** (K^+) across an NC700 membrane. (A) 1.0 M KOH. (B) 1.0 M KCl.

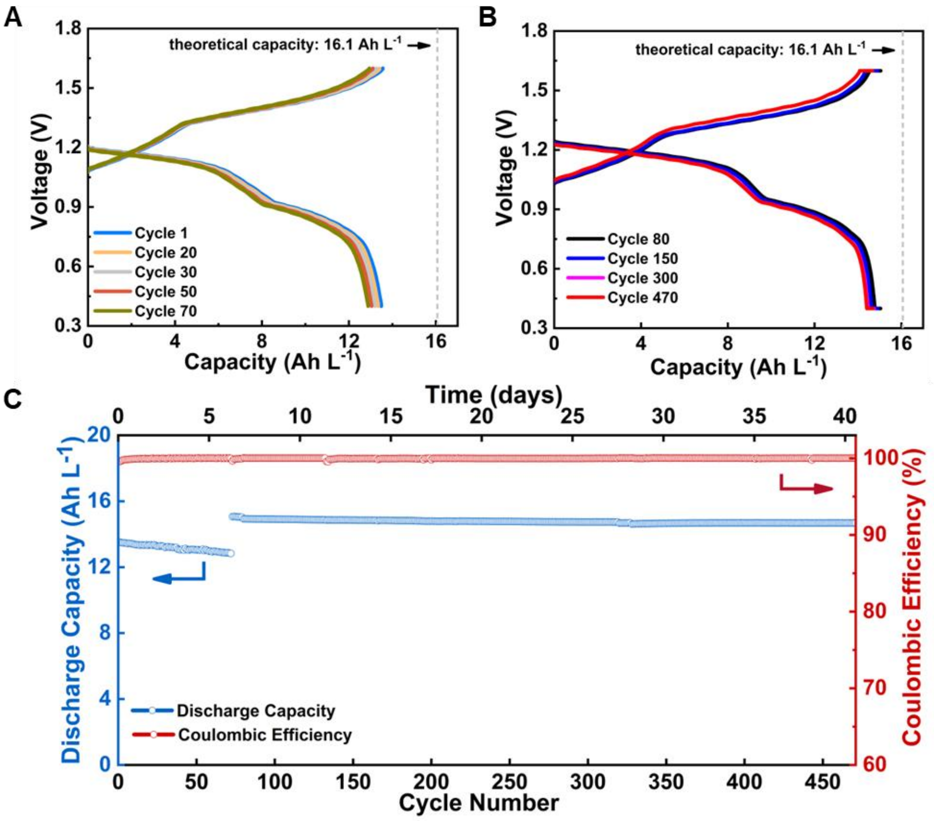

Figure S16 Cycling performance of 0.1 M **PPA** (K^+) in 1.0 M KCl. Electrolytes comprised 7 mL of 0.1 M **PPA** (K^+) (negolyte) in 1.0 M KCl, and 50 mL of 0.15 M $K_4Fe(CN)_6$ and 0.01 M $K_3Fe(CN)_6$ in 1.0 M KCl (posolyte). The cell was applied with a galvanostatic cycling at 20 mA cm^{-2} between 1.6 V and 0.4 V for 5.0 days, and continuously a galvanostatic-potentiostatic cycling for 35.6 days, while each half-cycle ended with a potentiostatic hold until the magnitude of the current density fell below 4 mA cm^{-2}. (A) Galvanostatic charge-discharge voltage profile from selected cycles. (B) Galvanostatic-potentiostatic charge-discharge voltage profile from selected cycles. (C) Coulombic efficiency and discharge capacity versus time and cycle numbers.

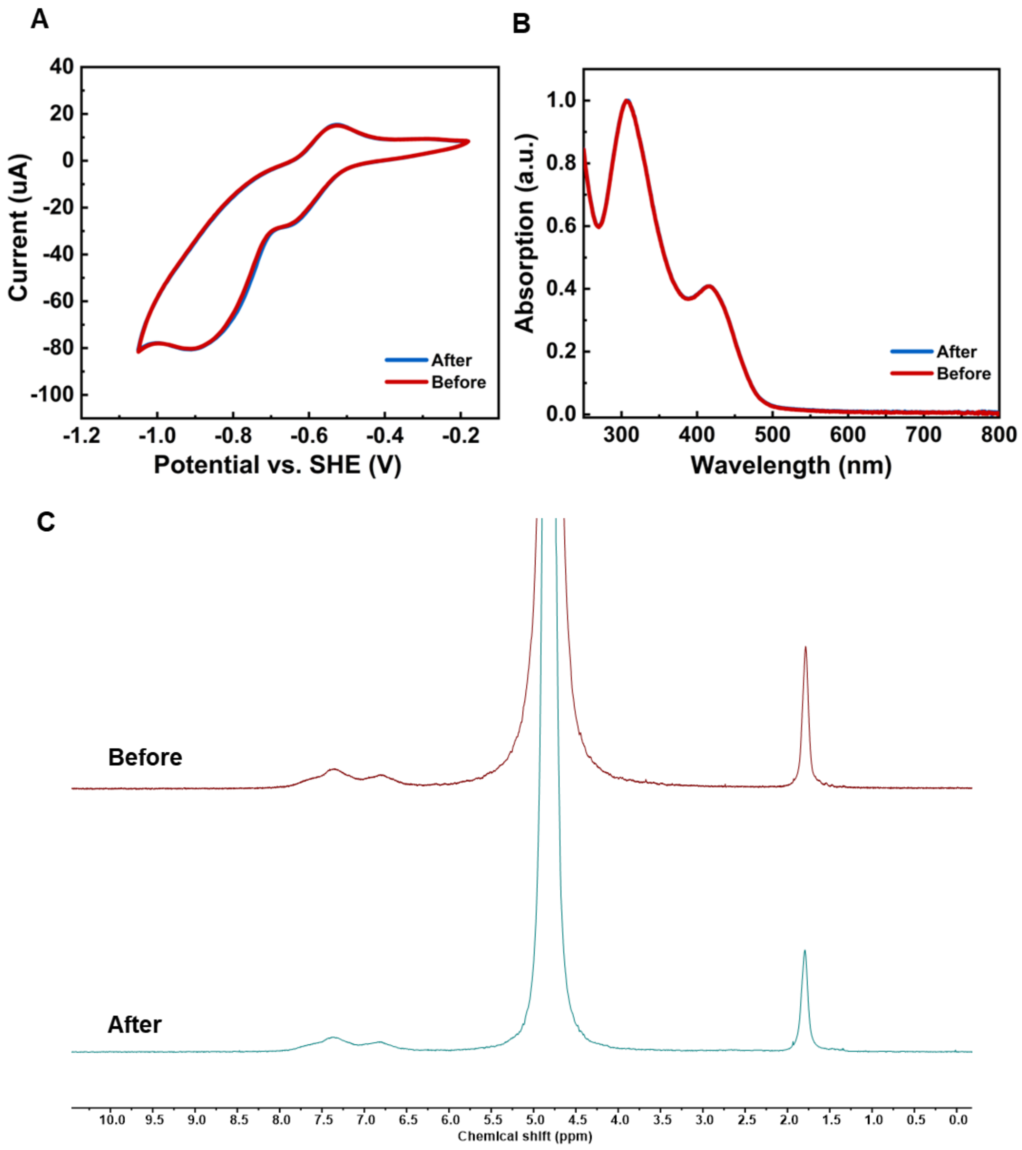

Figure S17 Post-cycling analysis of 0.1 M **PPA** (K^+) cell in 1.0 M KCl before and after 471 cycles. (A) CV trace of **PPA** before and after cell cycling. For CV analysis, 0.5 mL of the **PPA** electrolyte before and after cycling was collected and diluted to 1.5 mL 1.0 M KCl. The scan rate was 20 mV s^{-1}. (B) UV-vis spectra of **PPA** before and after cell cycling. (C) The ^{1}H NMR spectra of **PPA** before and after cell cycling. Samples for NMR analysis were diluted in D_2O at a fixed volume ratio of 1:4.

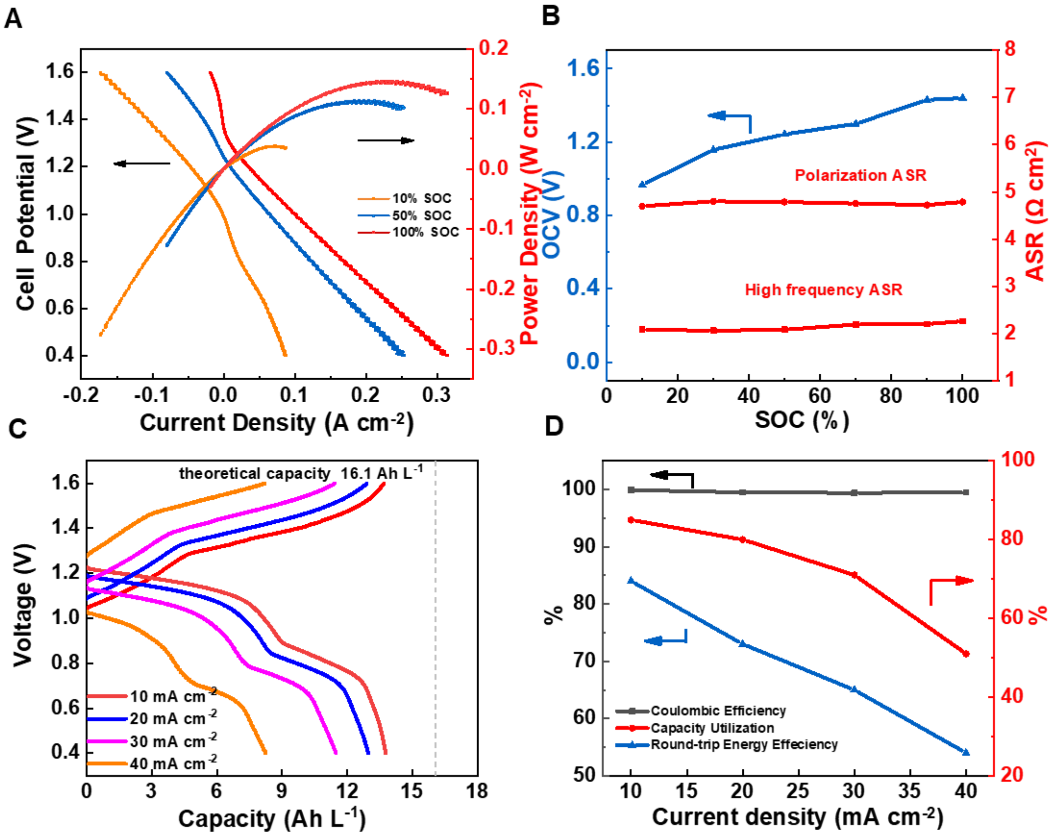

Figure S18 Polarization measurements of 0.1 M **PPA** (K^+) in 1.0 M KCl. Electrolytes comprised 7 mL of 0.1 M **PPA** (K^+) (negolyte) in 1.0 M KCl, and 50 mL of 0.15 M $K_4Fe(CN)_6$ and 0.01 M $K_3Fe(CN)_6$ in 1.0 M KCl (posolyte) with NC700 as exchange membrane. (A) Cell potential and power density versus current density at 10%, 50% and 100% SOC. (B) Full cell OCV, high-frequency, and polarization ASR versus various SOCs. (C) The capacity and galvanostatic charge-discharge voltage profiles at different current densities, the vertical dashed line indicates the theoretical capacity. (D) Capacity utilization, Coulombic efficiency, and round-trip energy efficiency versus current density.

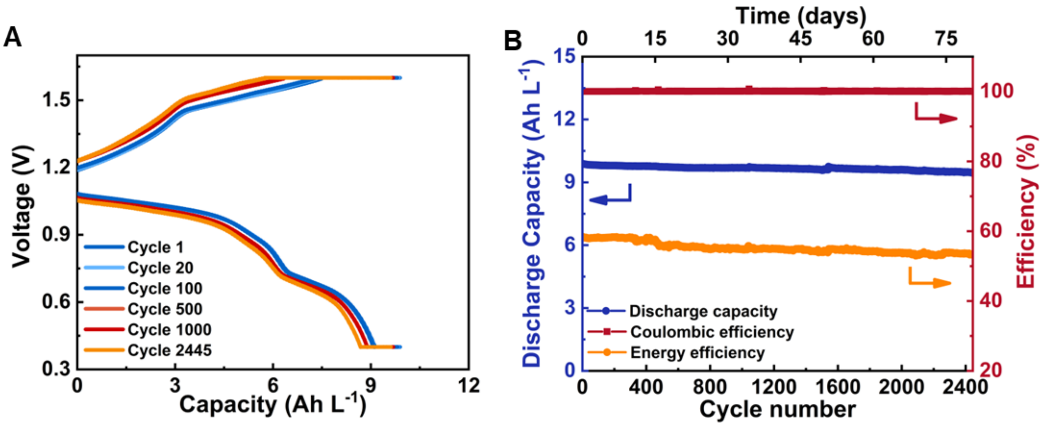

Figure S19 Cycling Performance of 0.1 M **PPA** (K^+) in 1.0 M KCl. Electrolytes comprised 7 mL of 0.1 M **PPA** (K^+) (negolyte) in 1.0 M KCl, and 50 mL of 0.15 M $K_4Fe(CN)_6$ and 0.01 M $K_3Fe(CN)_6$ in 1.0 M KCl (posolyte). The cell was cycled galvanostatic-potentiostatic at 40

mA cm^{-2} between 1.6 and 0.4 V and each half-cycle ended with a potentiostatic hold until the magnitude of the current density fell below 4 mA cm^{-2} for approximately 2445 cycles, 80.5 days. (A) Galvanostatic-potentiostatic charge-discharge voltage profile from selected cycles; (B) Coulombic efficiency, energy efficiency, and discharge capacity versus time and cycle numbers.

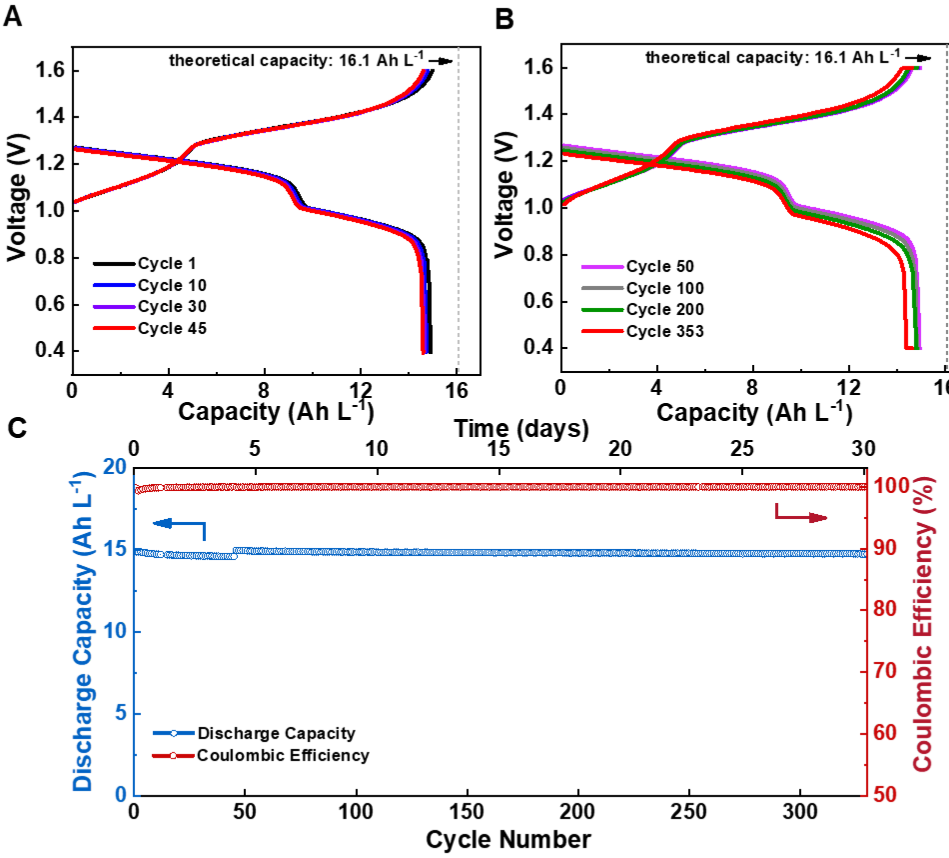

Figure S20 Cycling performance of 0.1 M **PPA** (K^+) in 1.0 M KOH. Electrolytes comprised 7 mL of 0.1 M **PPA** (K^+) (negolyte) in 1.0 M KOH, and 50 mL of 0.13 M $K_4Fe(CN)_6$ and 0.01 M $K_3Fe(CN)_6$ in 1.0 M KOH (posolyte). The cell was applied with a galvanostatic cycling at 20 mA cm^{-2} between 1.6 V and 0.4 V for 3.7 days, and continuously a galvanostatic-potentiostatic cycling for 26.4 days, while each half-cycle ended with a potentiostatic hold until the magnitude of the current density fell below 4 mA cm^{-2}. (A) Galvanostatic charge-discharge voltage profile from selected cycles. (B) Galvanostatic-potentiostatic charge-discharge voltage profile from selected cycles. (C) Coulombic efficiency and discharge capacity versus time and cycle number.

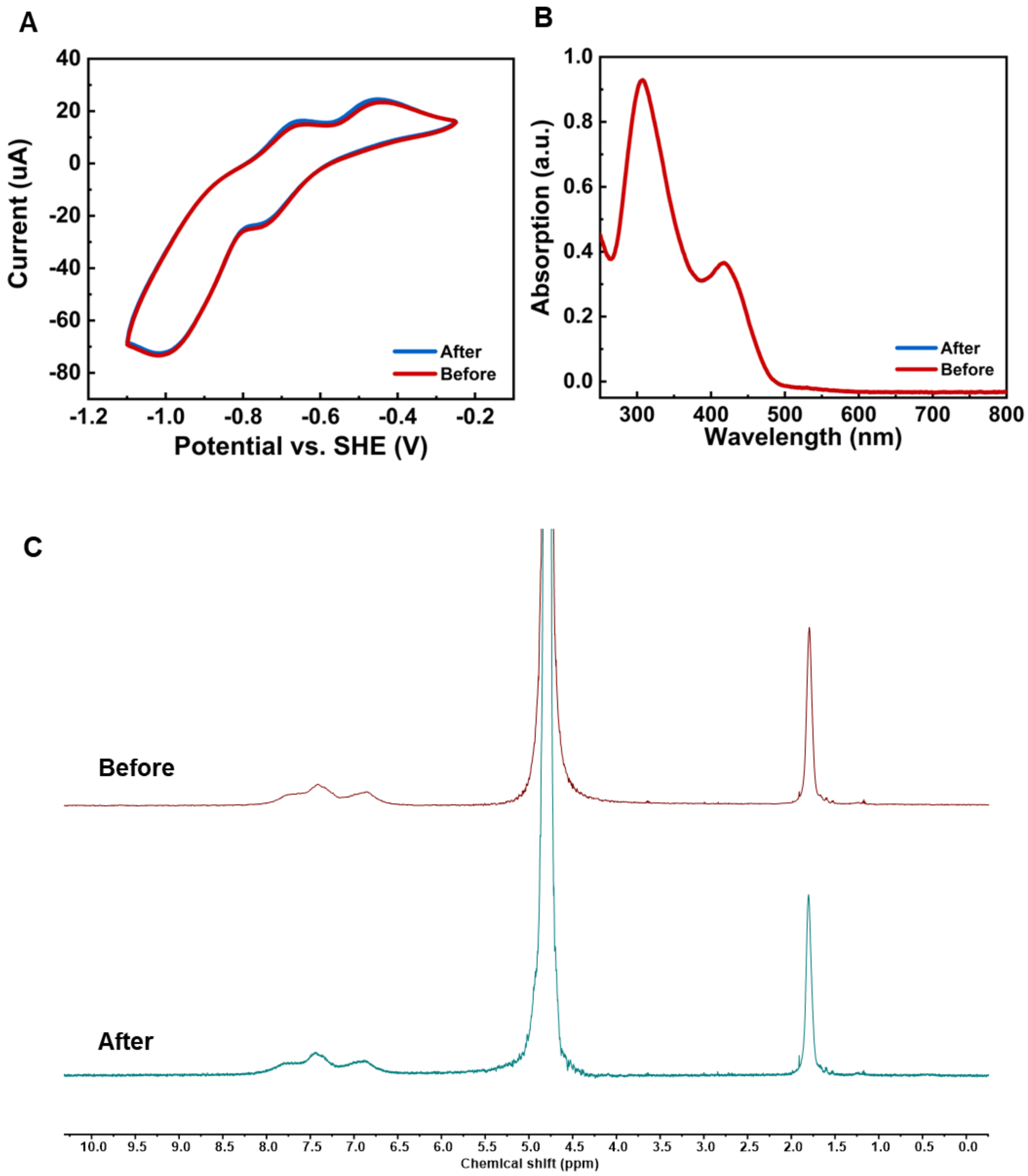

Figure S21 Post-cycling analysis of 0.1 M **PPA** (K^+) cell in 1.0 M KOH before and after 353 cycles. (A) CV trace of **PPA** before and after cell cycling. For CV analysis, 0.5 mL of the **PPA** electrolyte before and after cycling was collected and diluted to 1.5 mL 1.0 M KOH. The scan rate was 20 mV s^{-1}. (B) UV-vis spectra of **PPA** before and after cell cycling. (C) The ^1H NMR spectra of **PPA** before and after cell cycling. Samples for NMR analysis were diluted in D_2O at a fixed volume ratio of 1:4.

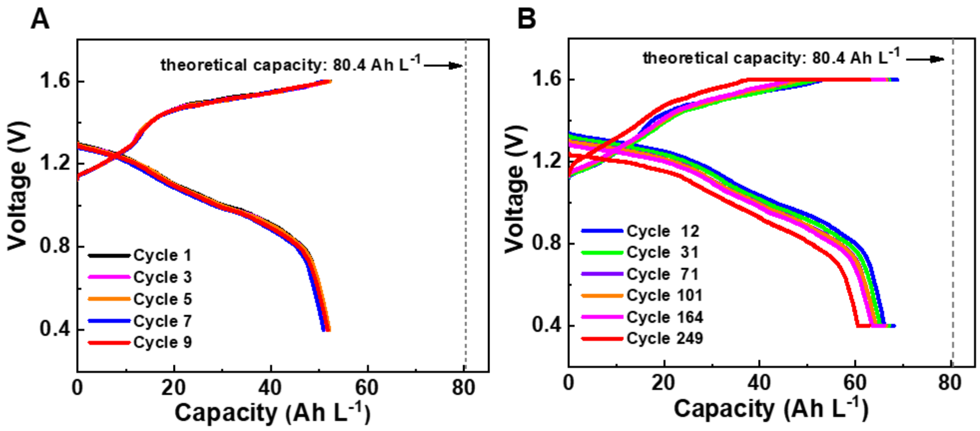

Figure S22 Cycling performance of 0.5 M **PPA** (K^+) in H_2O. Electrolytes comprised 5 mL of 0.5 M **PPA** (K^+) (negolyte) in H_2O, and 20 mL of 0.4 M $K_4Fe(CN)_6$, 0.6 M $Na_4Fe(CN)_6$ and 0.4 M $K_3Fe(CN)_6$ in H_2O (posolyte). The cell was applied with a galvanostatic cycling at 20 mA cm^{-2} between 1.6 V and 0.4 V for 2.3 days, and continuously a galvanostatic-potentiostatic cycling for 77.7 days, while each half-cycle ended with a potentiostatic hold until the magnitude of the current density fell below 4 mA cm^{-2}. (A) Galvanostatic charge-discharge voltage profile from selected cycles. (B) Galvanostatic-potentiostatic charge-discharge voltage profile from selected cycles.

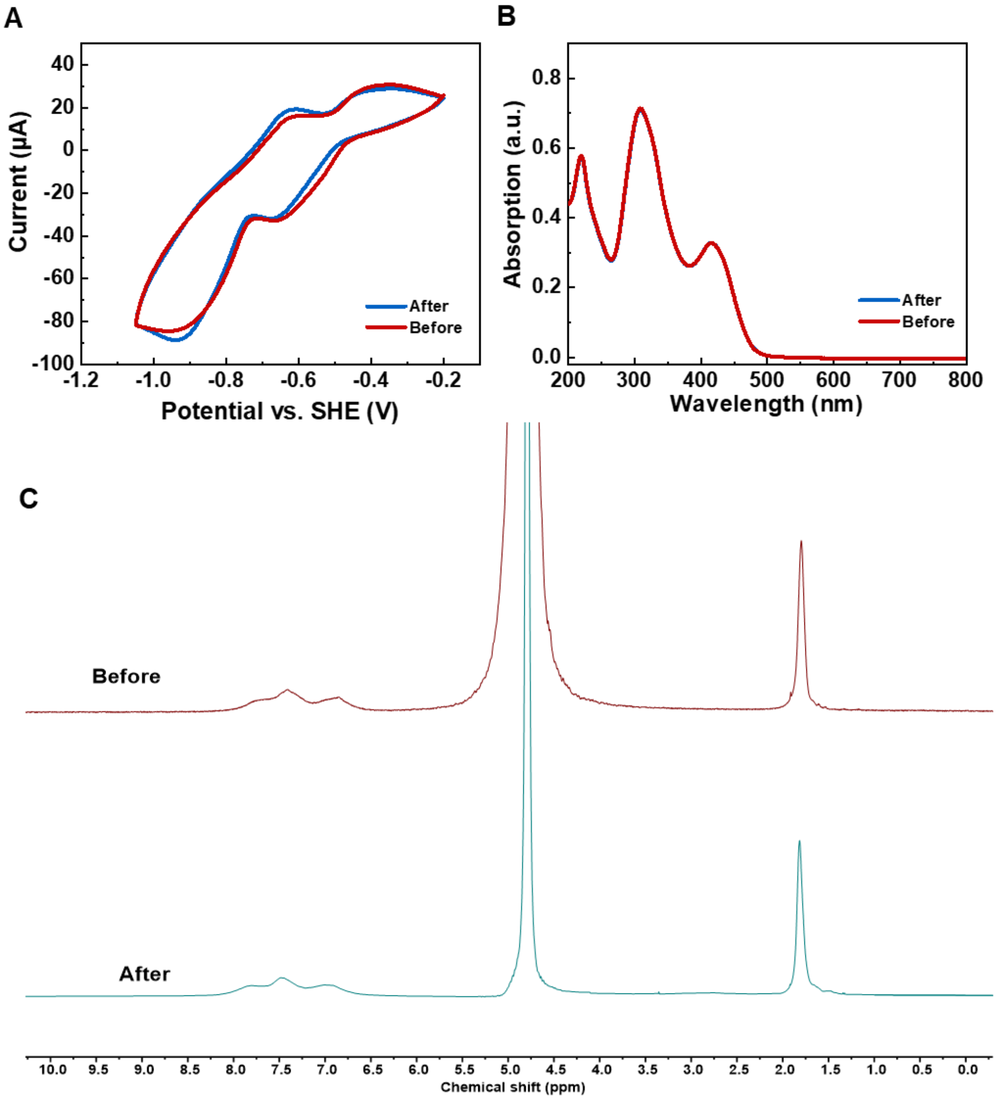

Figure S23 Post-cycling analysis of 0.5 M **PPA** (K^+) cell in H_2O before and after 236 cycles. (A) CV trace of **PPA** before and after cell cycling. For CV analysis, 0.1 mL of the **PPA** electrolyte before and after cycling was collected and diluted into 1.9 mL 1.0 M KCl. The scan rate was 20 mV s^{-1}. (B) UV-vis spectra of **PPA** before and after cell cycling. (C) The ^{1}H NMR spectra of **PPA** before and after cell cycling. Samples for NMR analysis were diluted in D_2O at a fixed volume ratio of 1:20.

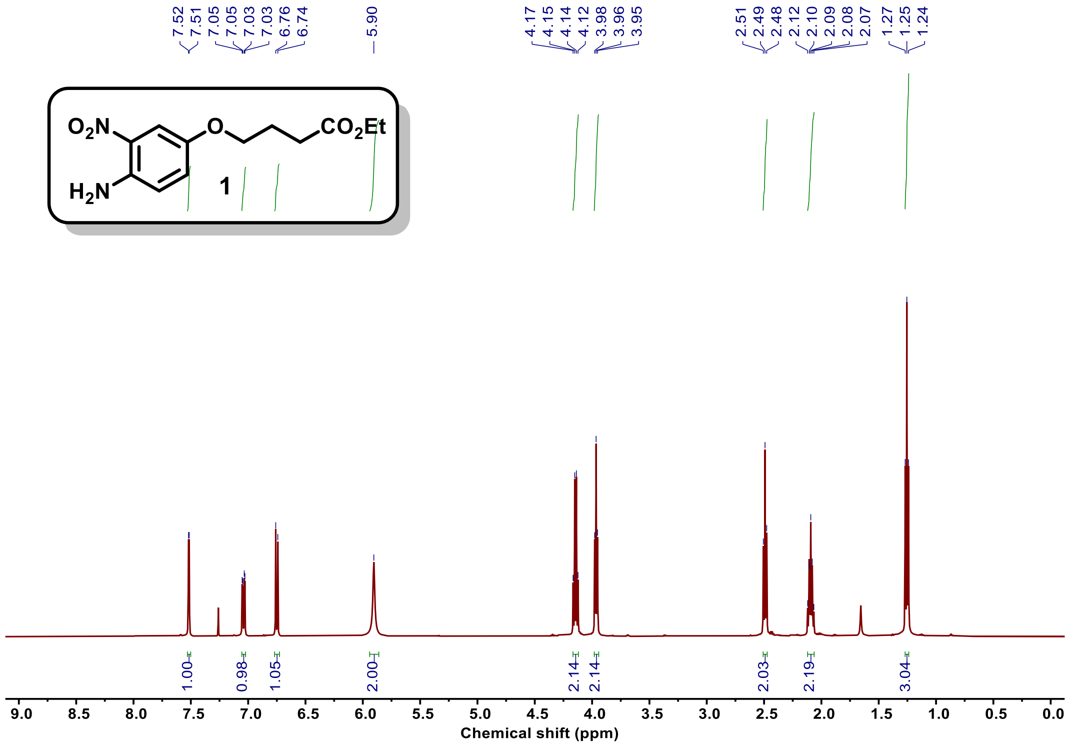

Figure S24 ^{1}H NMR spectra of **1** ($CDCl_3$, 500 MHz).

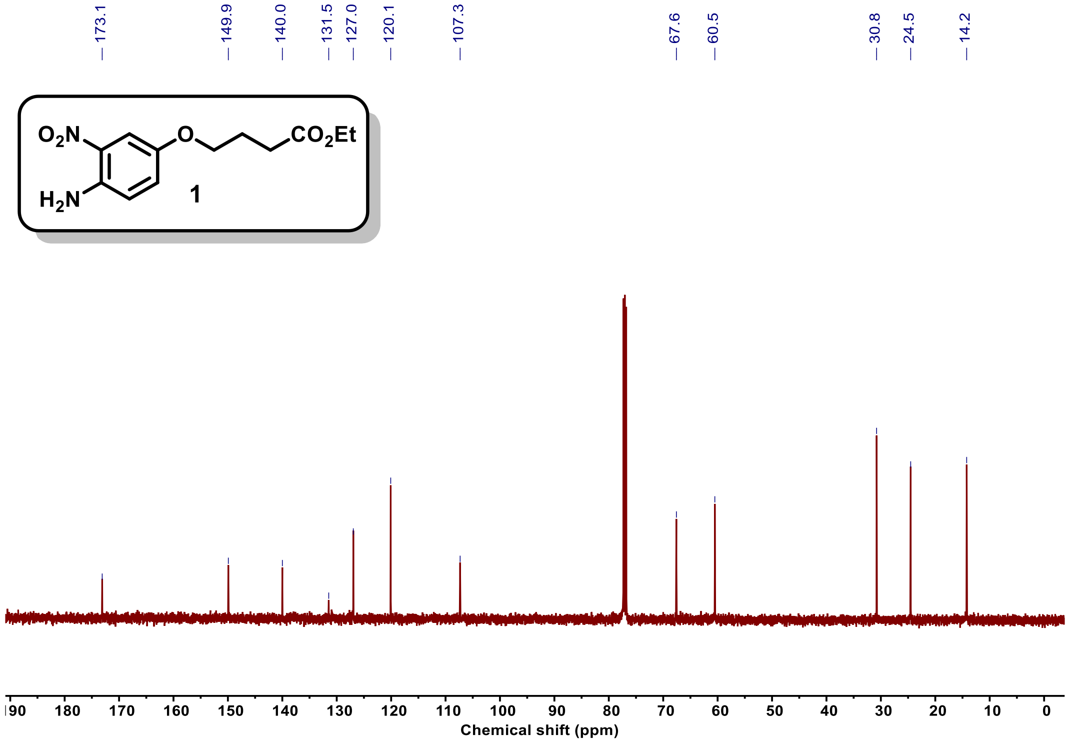

Figure S25 ^{13}C NMR spectra of **1** ($CDCl_3$, 125 MHz).

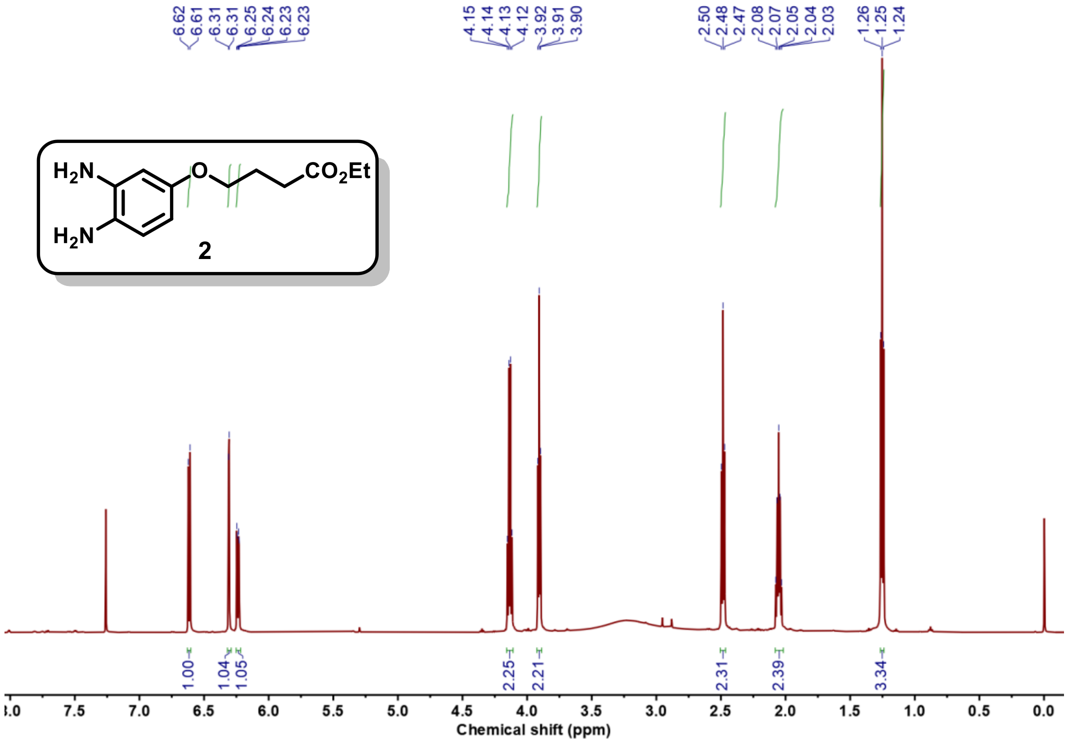

Figure S26 ^{1}H NMR spectra of **2** ($CDCl_3$, 500 MHz).

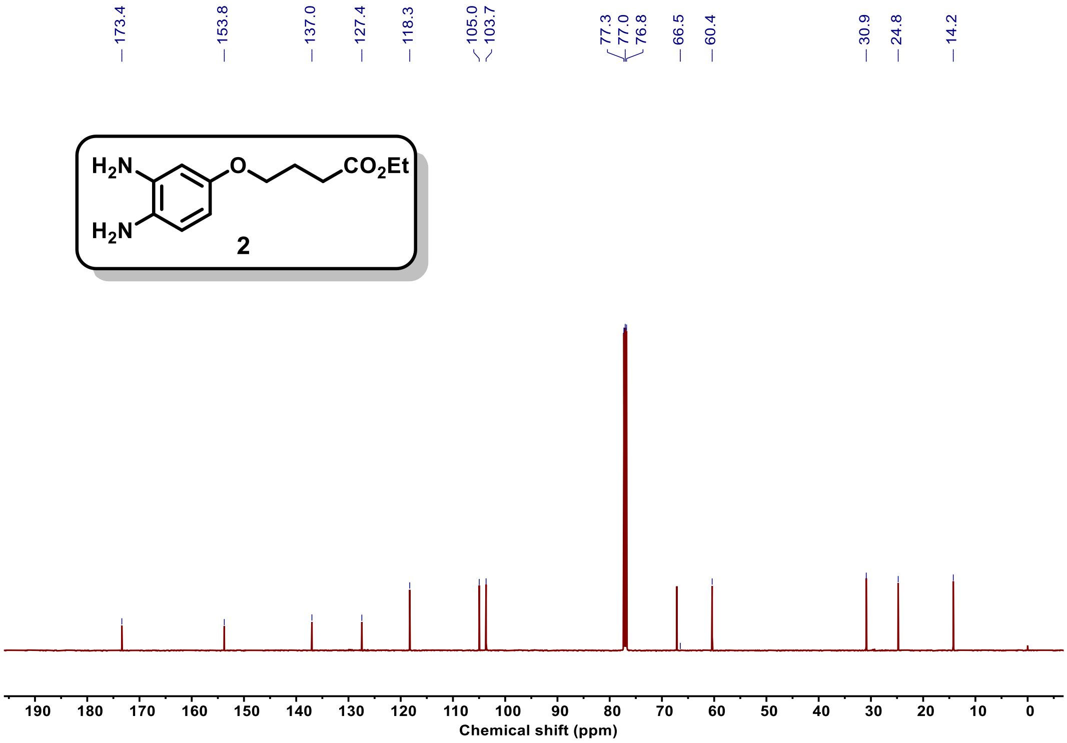

Figure S27 ^{13}C NMR spectra of **2** ($CDCl_3$, 125 MHz).

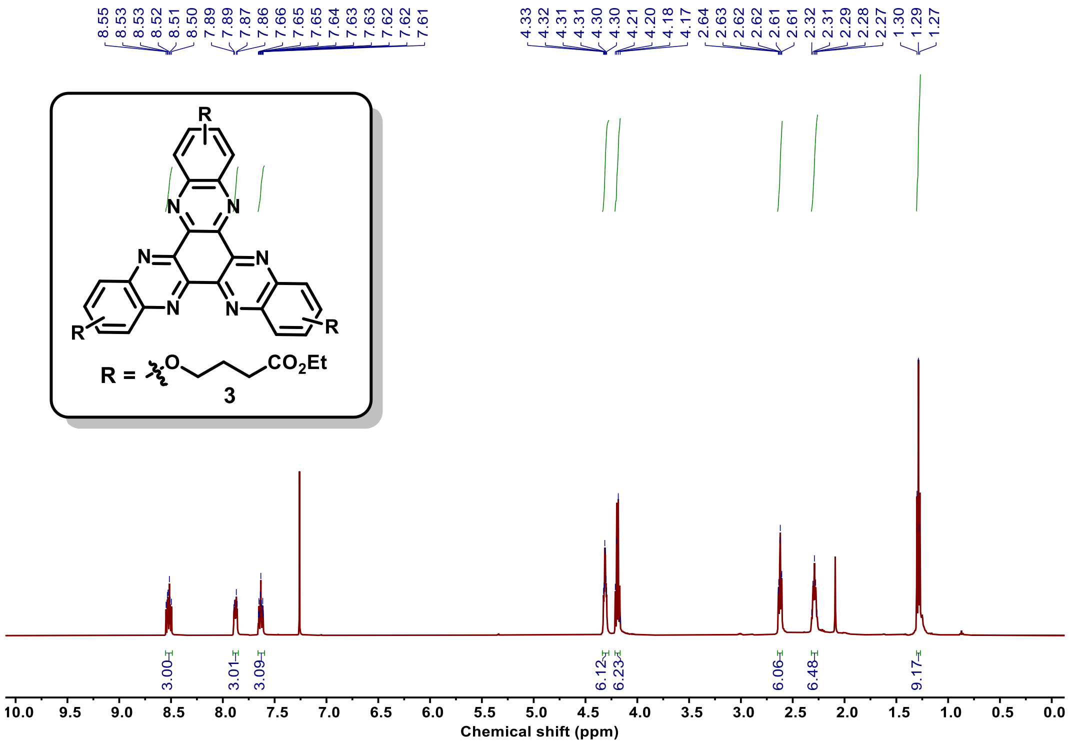

Figure S28 ^{1}H NMR spectra of **3** ($CDCl_3$, 500 MHz).

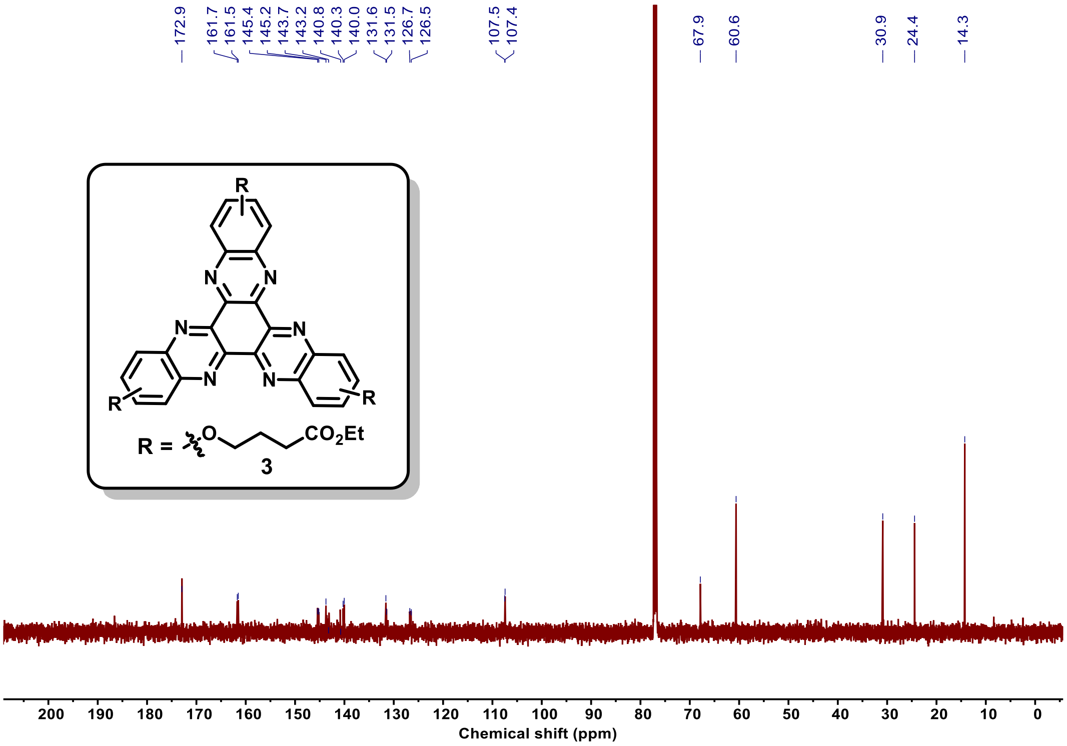

Figure S29 ^{13}C NMR spectra of **3** ($CDCl_3$, 125 MHz).

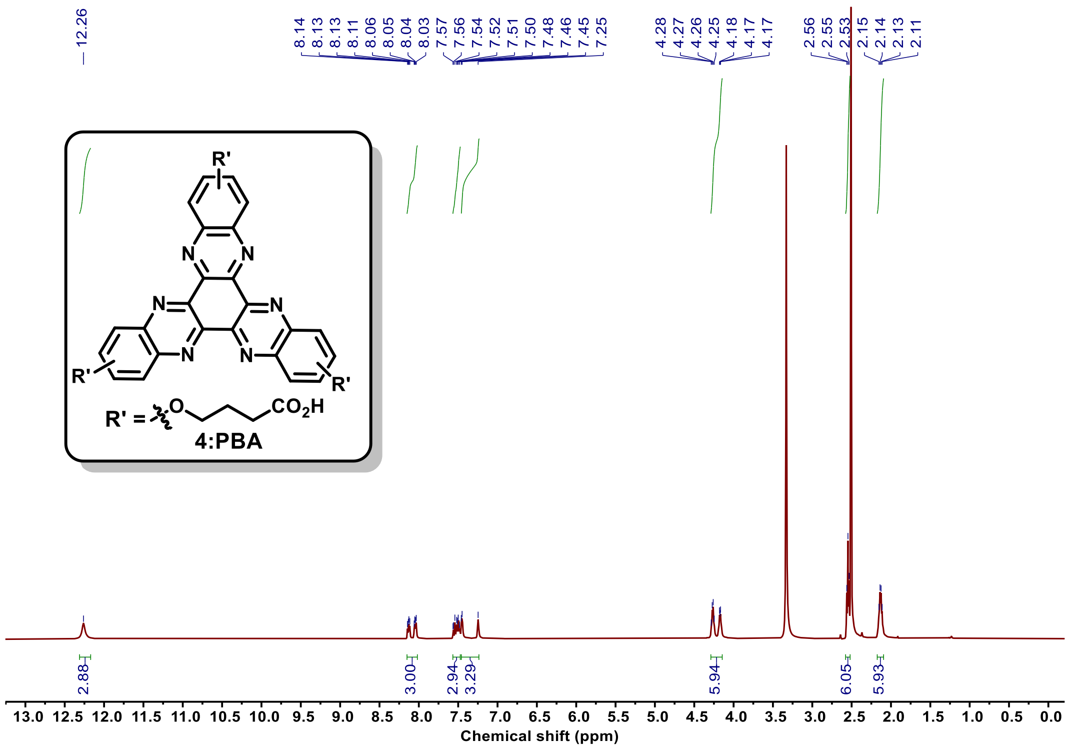

Figure S30 ^{1}H NMR spectra of **PBA** (DMSO-d_6, 500 MHz).

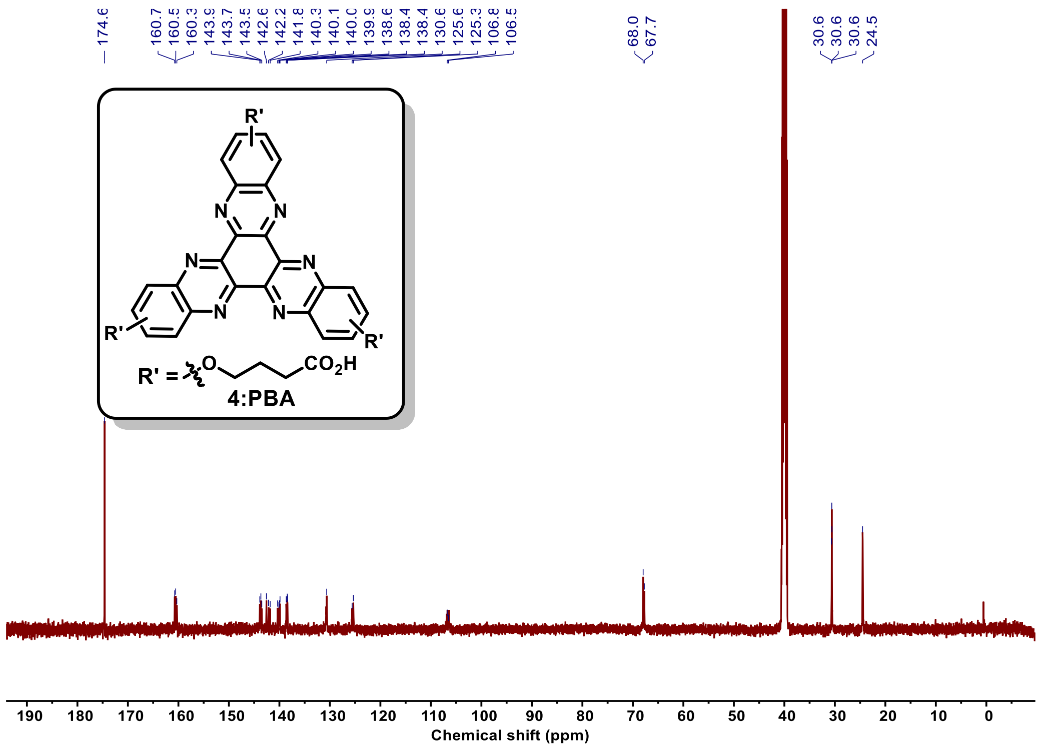

Figure S31 ^{13}C NMR spectra of **PBA** (DMSO-d_6, 125 MHz).

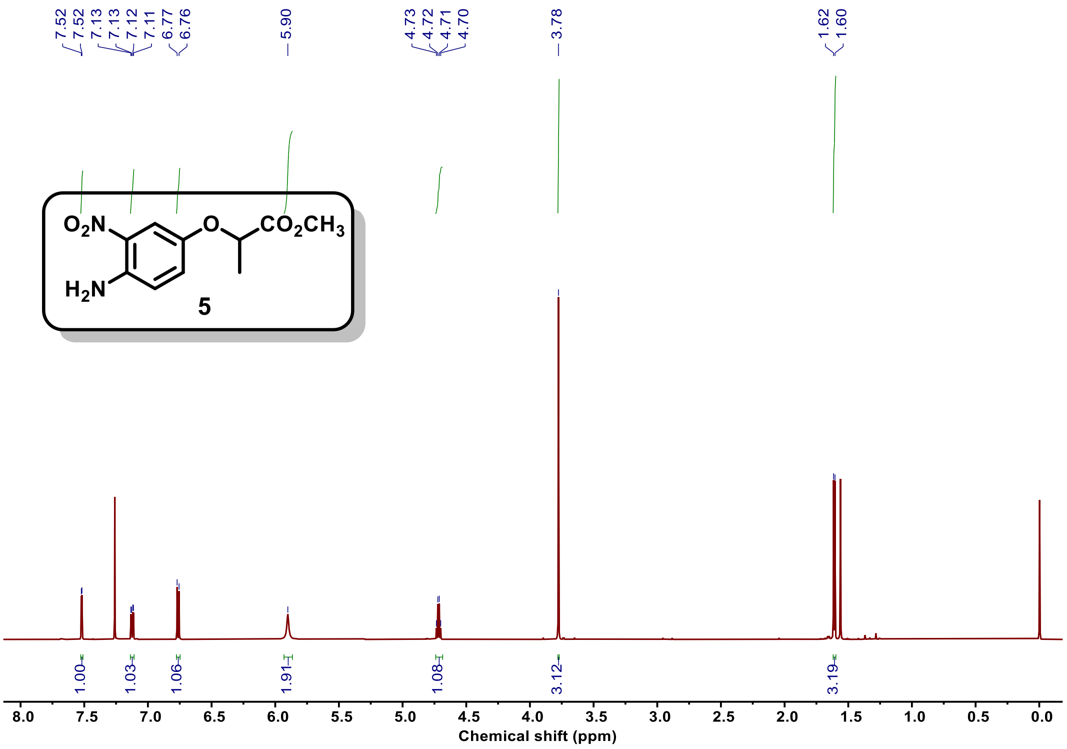

Figure S32 ^{1}H NMR spectra of **5** ($CDCl_3$, 500 MHz).

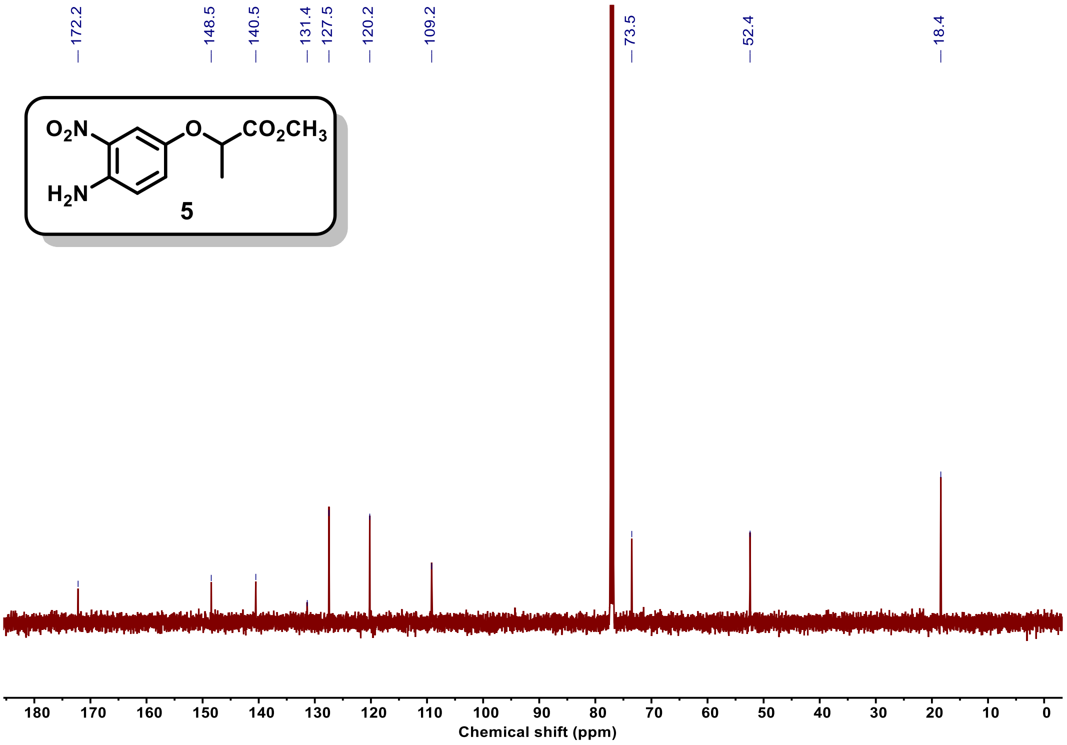

Figure S33 ^{13}C NMR spectra of **5** ($CDCl_3$, 125 MHz).

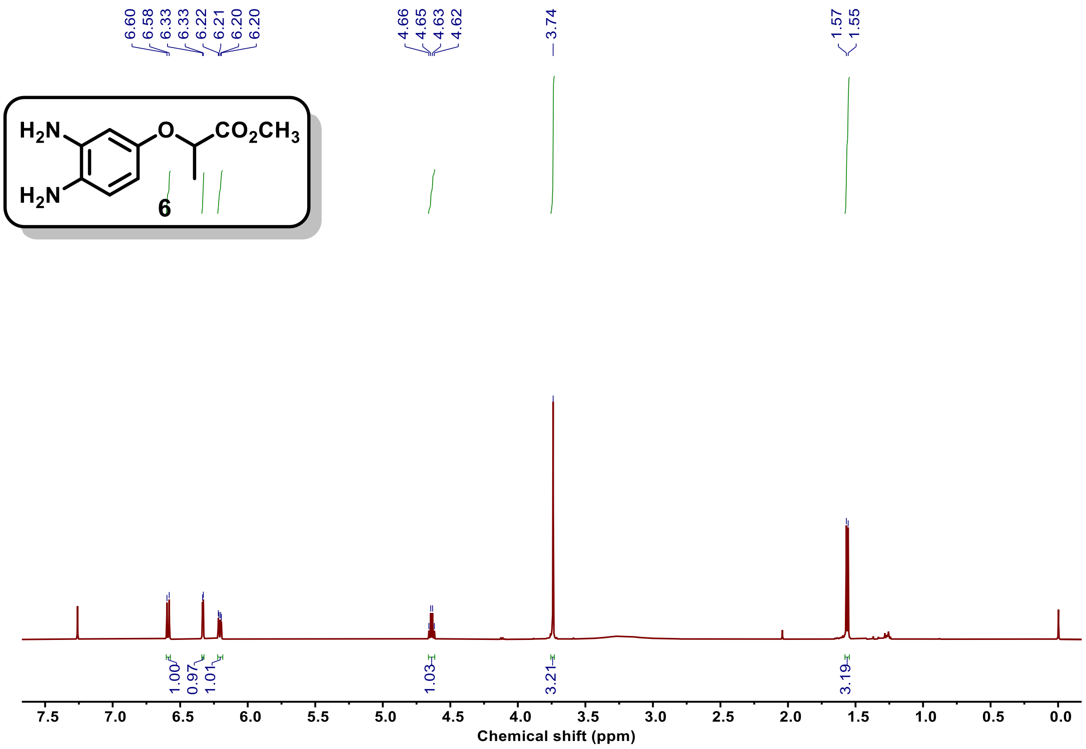

Figure S34 ^{13}C NMR spectra of **6** ($CDCl_3$, 500 MHz).

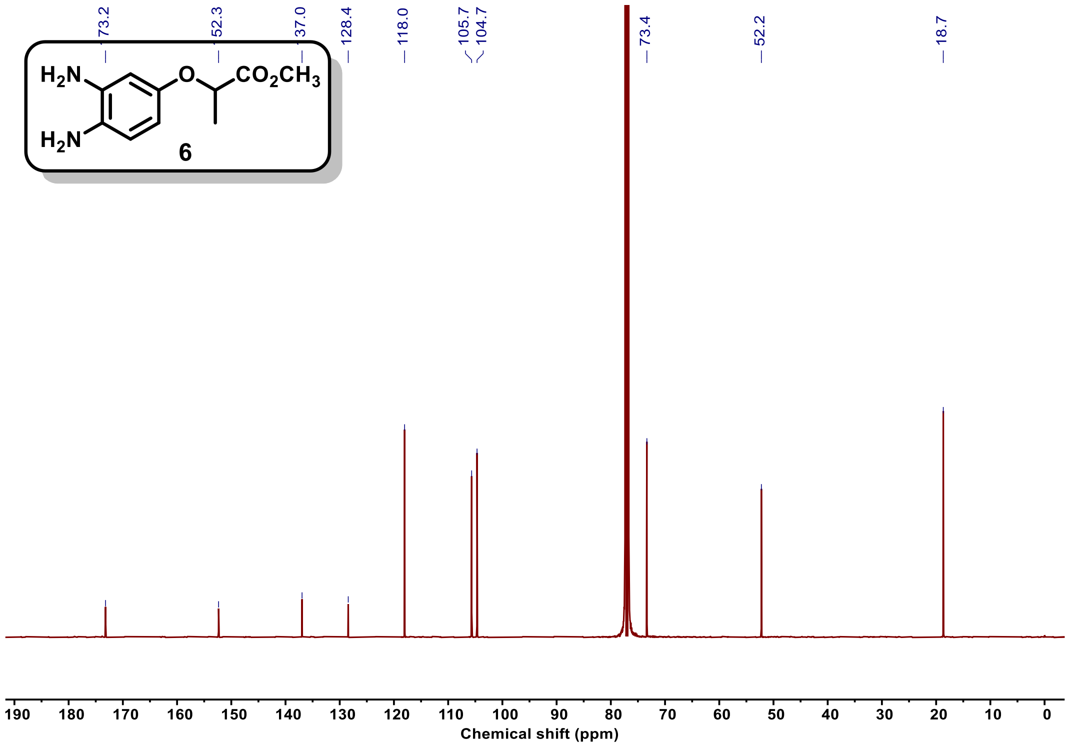

Figure S35 ^{13}C NMR spectra of **6** ($CDCl_3$, 125 MHz).

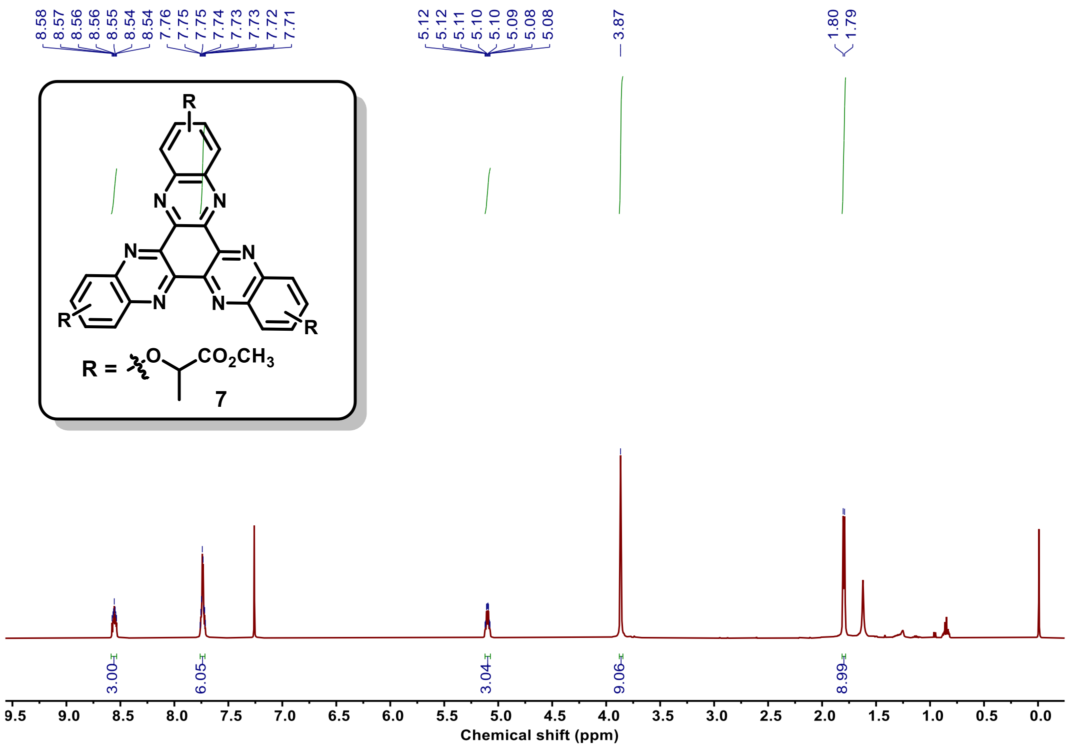

Figure S36 ^{1}H NMR spectra of **7** ($CDCl_3$, 500 MHz).

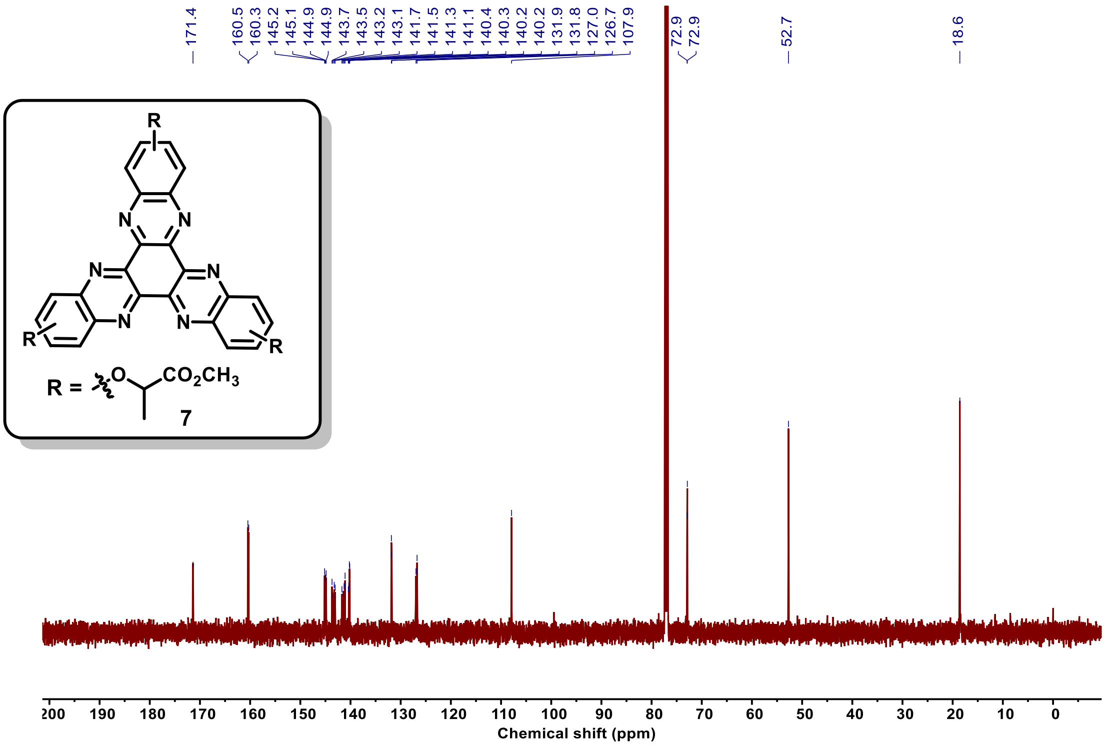

Figure S37 ^{13}C NMR spectra of **7** ($CDCl_3$, 125 MHz).

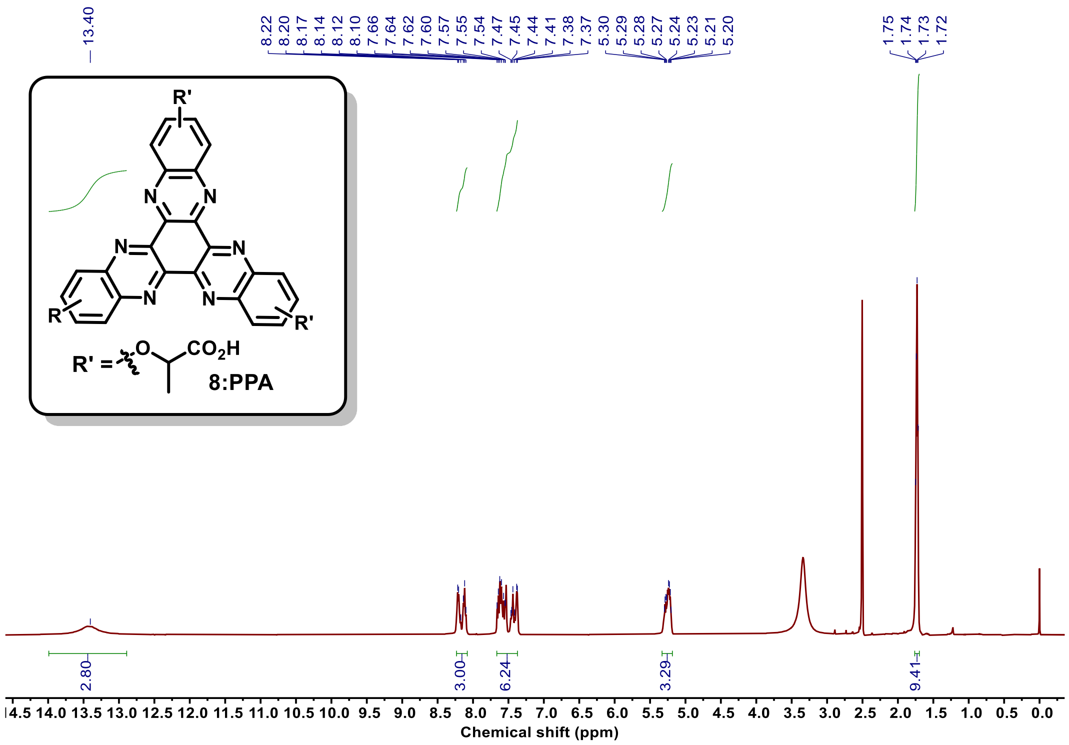

Figure S38 ^{1}H NMR spectra of **PPA** (DMSO-d_6, 500 MHz).

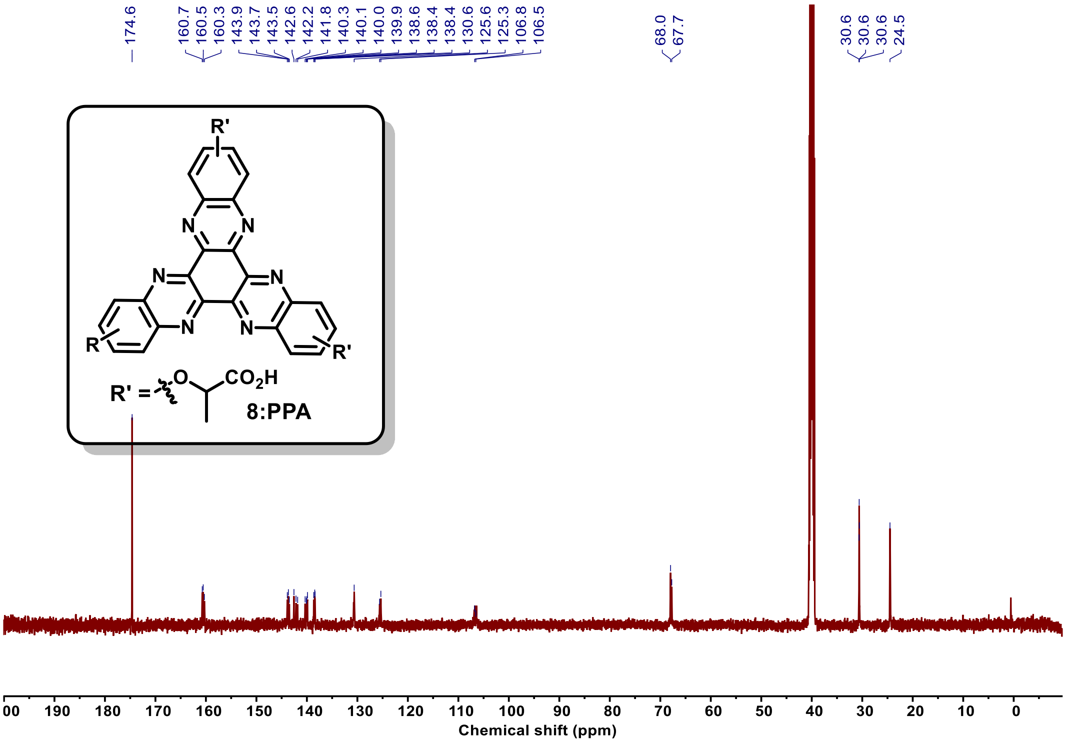

Figure S39 ^{13}C NMR spectra of **PPA** (DMSO-d_6, 125 MHz).

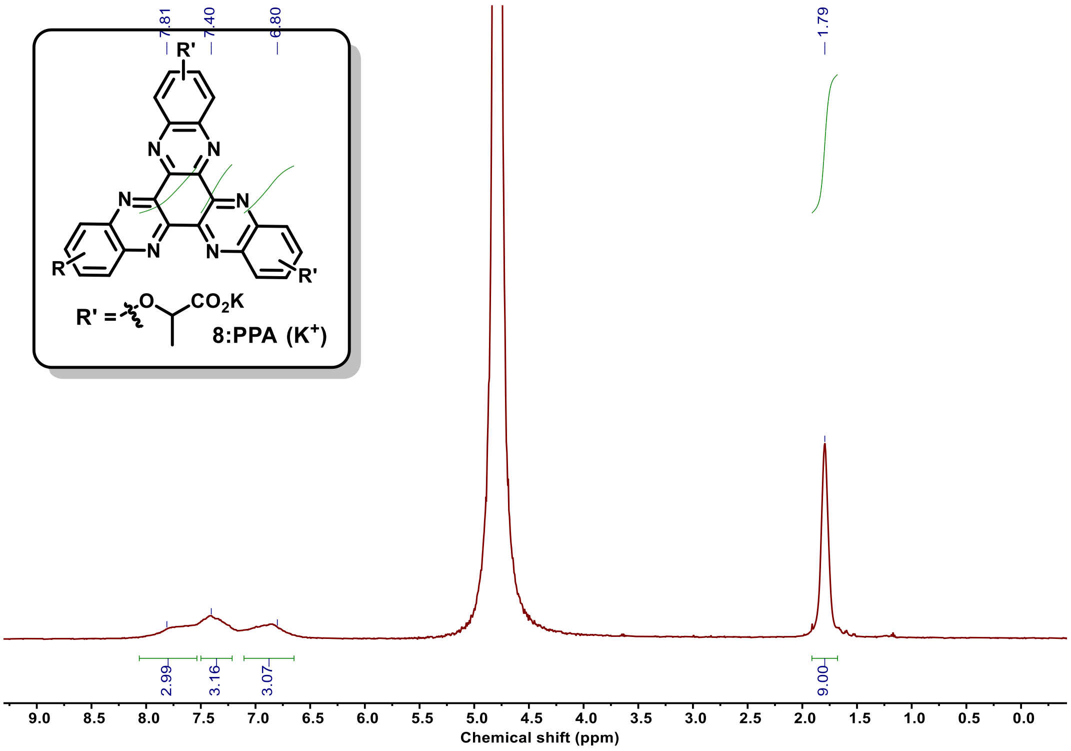

Figure S40 ^{1}H NMR spectra of **PPA** (K^+) (D_2O, 500 MHz).

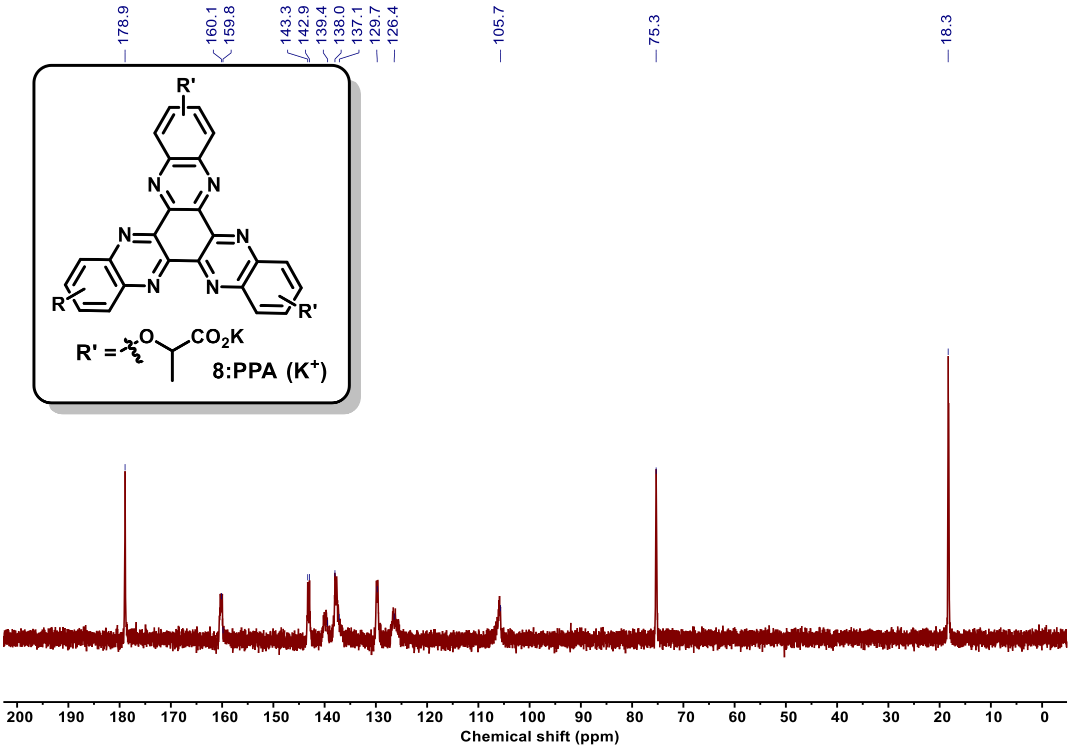

Figure S41 ^{13}C NMR spectra of **PPA** (K^+) (D_2O, 125 MHz).

Table S1 The calculation to compare the solvation energy of **PPA** and **PBA**.

	ΔG_{solv} (kcal mol^{-1})
**PBA**	- 276.94
**PPA**	- 283.86

Table S2 Summary table of molecular formulas in different ball-and-stick model representations.

	**Ball and stick model**	**Molecular formulas**
**Int 1-1**	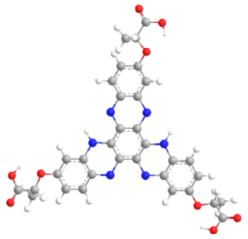	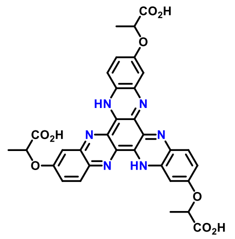
**Int 1-2**	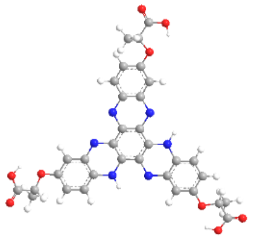	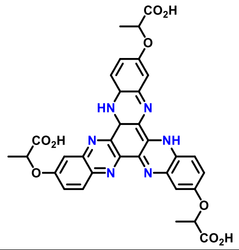
**Int 1-3**	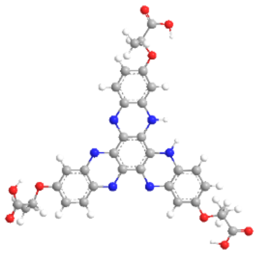	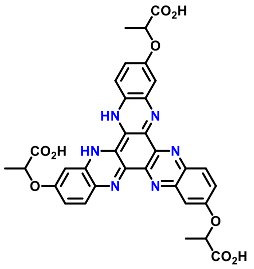
**Int 1-4**	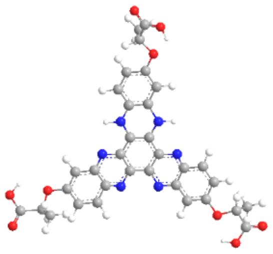	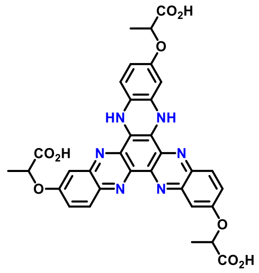

	Ball and stick model	Molecular formulas
**Int 2-1**	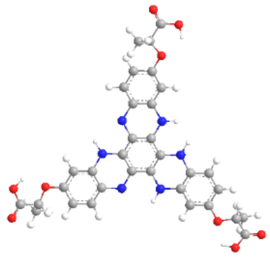	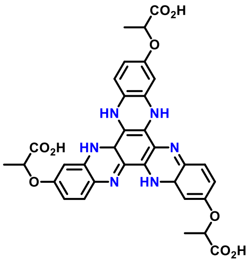
**Int 2-2**	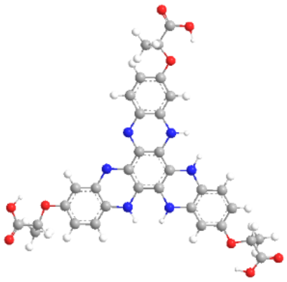	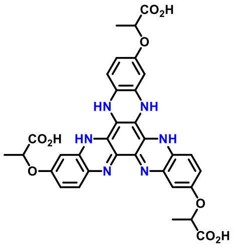
**Int 2-3**	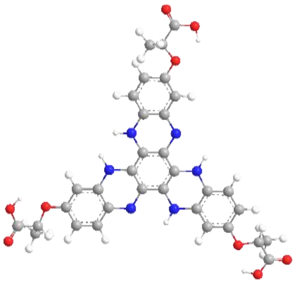	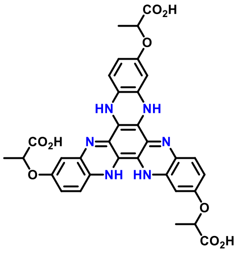
**Int 2-4**	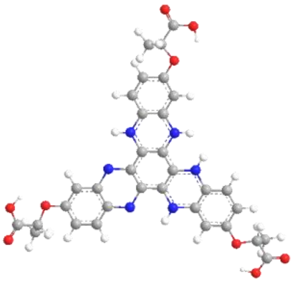	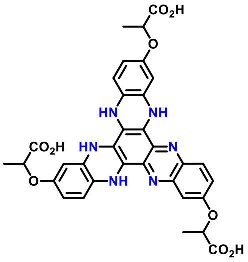

Table S3 Comparison of the performance of various multiple negolyte redox materials in aqueous organic redox flow batteries.

Negolyte		Solubility max	Power density (mW/cm²)	Energy efficiency (%)	Capacity (Ah/L)	Cycle numbers/ days	Capacity fade rate (%) per cycle/per day	Ref.
**DHBQ**	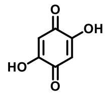	4.31	300	88	23.15	150/NA	0.24/NA	*Adv. Energy Mater.* **2018**, 8, 1702056
**DHAQ**	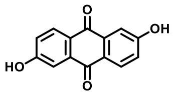	0.6	700	84	26.8	100/NA	0.1/NA	*Science* **2015**, 349,1529-1532.
**2,6-DBEAQ**	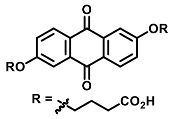	1.1	240	80	34.8	250/5	0.001/0.05	*Joule*, **2018**, 2, 1894-1906
**FMN-Na**	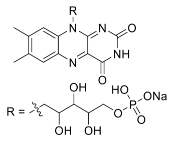	1.5	160	60	12.86	200/NA	0.01/NA	*Nat. Commun.* **2016**, 7, 13230.
**AQDS(NH₄)₂**	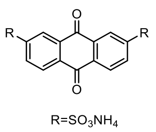	1.9	91.5	70	12.5	300/15	0	*Angew. Chem. Int. Ed.* ***2019****, 58,* 16629-16636.
**DCDHAQ**	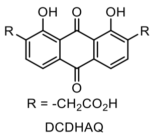	1.3	160	NA	40.2	90/9	0.0029/0.03	*J. Mater. Chem. A,* ***2021****, 9, 26709-26716.*
**NAPD**	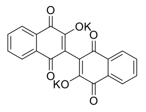	0.56	280	79	51.2	250/12.8	0.038/0.74	*ACS Energy Lett.* **2019**, 4, 1880-1887.
**AMA**	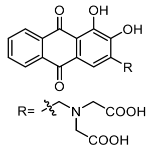	0.4	490	84.9	6.1	350/NA	NA/NA	*ACS Appl. Energy Mater.* **2019**, 2, 2469-2474.
**AHP**	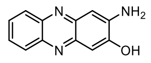	0.43	NA	80	4.39	100/NA	NA/NA	*ACS Energy Lett.* **2020**, 5, 411-417.
**1,8-PFP**	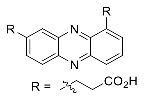	2.05	55	80	53.6	345/53	0/0	*Joule* **2021**, 5, 2437-2449.
**1,8-ESP**	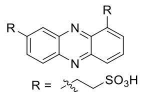	2.06	150	86	50.0	1125/180	0.008/0.05	*Nat. Energy*, *2023*, 8 1126-1136.

**TPz-2**	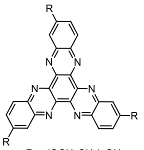	0.3	NA	81	0.80	50/NA	0.6/55	*Chem. Commun.* **2022**, 58, 13226-13229.
**HATNTA**	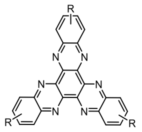	0.583	238	NA	37.2	80/10	0.021/0.168	*Energy Storage Mater. **2023**, 59, 102789.*

Table S4 Summary of the electrochemical properties and capacity fade rate of 6e⁻ materials

Negolyte	D ($cm^2\ s^{-1}$)	k_0 (cm s^{-1})	Capacity (Ah L^{-1})	Cycle numbers/ days	Capacity fade rate (%) per cycle/per day	Ref.
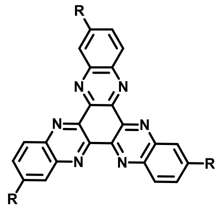 R = -($(OCH_2CH_2)_3OH$ **TPz-2**	3.16×10^{-8} 8.88×10^{-8}	1.13×10^{-5} 1.69×10^{-5}	0.48	50/0.69	0.6/55	*Chem. Commun.*, **2022**, 58, 13226-13229
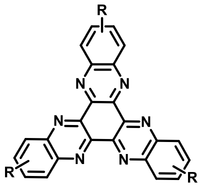 R' = -COOH **HATNTA**	2.43×10^{-6}	2.32×10^{-3}	37.2	80/10	0.21/0.168	*Energy Storage Mater.* **2023**, 59, 102789
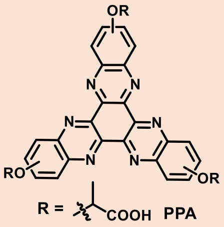 **PPA**	1.31×10^{-7} 1.65×10^{-7}	6.01×10^{-5} 1.29×10^{-4}	15.1	[a] 471/40.6	[a] 0.006/0.07	**1.0 M KCl (This work)**
			9.9	[b] 2445/80.5	[b] 0.0017/0.05	**1.0 M KCl (This work)**
			15.0	[c] 353/30.1	[c] 0.007/0.09	**1.0 M KOH (This work)**
			68.2	[d] 236/80.0	[d] 0.032/0.095	**H_2O (This work)**

[a] 0.1 M **PPA** (K^+) in 1.0 M KCl, cycled at 20 mA cm^{-2}; [b] 0.1 M **PPA** (K^+) in 1.0 M KCl, cycled at 40 mA cm^{-2}; [c] 0.1 M **PPA** (K^+) in 1.0 M KOH, cycled at 20 mA cm^{-2}; [d] 0.5 M **PPA** (K^+) in H_2O, cycled at 20 mA cm^{-2}.

Table S5 A summary of the batteries demonstrated.

Supporting electrolytes		1.0 M KCl	1.0 M KOH	H_2O
Solubility		1.1 M	0.8 M	1.2 M
Capacity (Ah/L)	theoretical	176.9	128.6	193.0
	demonstrated	15.1 (0.1 M **PPA** cell)	15.0 (0.1 M **PPA** cell)	68.2 (0.5 M **PPA** cell)
Power density (mW cm^{-2})		146	153	117
Coulombic efficiency (%)		> 99	> 99	> 99
Energy efficiency (%)		[a] 77, [b] 58	[a] 85	[a] 72
Capacity utilization (%)		[a] 94, [b] 61	[a] 93	[a] 85
Cycle numbers, days		[a] 471 cycles, 40.6 days<br>[b] 2445 cycles, 80.5 days	[a] 353 cycles, 30.1 days	[a] 236 cycles, 80.0 days
Capacity fade rate		[a] 0.006%/cycle, 0.07%/day<br>[b] 0.0017%/cycle, 0.05%/day	[a] 0.007%/cycle, 0.09%/day	[a] 0.032%/cycle, 0.095%/day

[a] Cycled at 20 mA cm^{-2}; [b] Cycled at 40 mA cm^{-2}.

Table S6 Viscosity measurements of **PPA** (K^+) with 0.1 M and 0.5 M concentrations.

**PPA** (K^+)		**Viscosity** η (Pa·s) [a]	
(M)	**T/ °C**	**0.1 M**	**0.5 M**
**In 1.0 M KOH**	RT	1.47×10^{-3}	0.16
	45 °C	1.03×10^{-3}	0.09
**In 1.0 M KCl**	RT	1.23×10^{-3}	0.13
	45 °C	0.89×10^{-3}	0.07
**In H_2O**	RT	1.11×10^{-3}	0.05
	45 °C	8.49×10^{-4}	0.03

[a] The measurements were conducted with 25.0 mm 1.0° stainless steel cone plate at room temperature and recorded on Rheometer TA-Waters ARES-G2 instrument. The viscosity values shown in the table are all under the same shear rate at 200 s^{-1}.

Supplementary references

[1] S. Pang, X. Y. Wang, P. Wang, Y. L Ji. *Angew. Chem. Int. Ed.* **2021**, *60*, 5289-5298.

[2] J. C. Xu, S. Pang, X. Y. Wang, P. Wang, Y. L. Ji. *Joule* **2021**, 5, 2437-2449.

[3] R. Evans, Z. Deng, A. K. Rogerson, A. S. McLachlan, J. J. Richards, M. Nilsson, G. A Morris. *Angew. Chem. Int. Ed.* **2013**, 52, 3199-3202.

[4] H. Wang, S. Y. Sayed, E. J. Luber, B. C. Olsen, S. M. Shirurkar, S. Venkatakrishnan, U. M.Tefashe, A. K. Farquhar, E. S. Smotkin, R. L. McCreery, J. M. Buriak. *ACS Nano*, **2020**, 14, 2575-2584.

[5] N. Elgrishi, K. J. Rountree, B. D. McCarthy, E. S. Rountree, T. T. Eisenhart, J. L. Dempsey. *J. Chem. Educ*. **2018**, 95, 197-206.

[6] A. D. Becke, *J. Chem. Phys.* **1993**, 98, 5648-5652.

[7] V. A. Rassolov, M. A. Ratner, J. A. Pople, P. C. Redfern, L. A. Curtiss. *J. Comput. Chem.* **2001**, 22, 976-984.

[8] R. Krishnan, J. S. Binkley, R. Seeger, J. A. Pople. *J. Chem. Phys.* **1980**, 72, 650-654.

[9] S. Grimme, J. Antony, S. Ehrlich, H. Krieg. *J. Chem. Phys.* **2010**, 132, 154104.

[10] A. V. Marenich, C. J. Cramer, D. G. Truhlar. *J. Phys. Chem. B* **2009**, 113, 6378-6396.

[11] J. Ho, A. Klamt, M. L. Coote. *J. Phys. Chem. A* **2010**, 114, 13442-13444.

[12] J. M. Wang, R. M. Wolf, J. W. Caldwell, P. A. Kollman, D. A. Case. *J. Comput. Chem.* **2004**, 25, 1157-1174.

[13] A. W. S. Silva, W. F. Vranken. *BMC Res Notes*. **2012**, 5, 367.

[14] S. Akiyama, S. Yamauchi, N. Hirota, S. Nagaoka. *J. Phys. Chem*. **1993**, 97, 40, 10269-10280.

[15] Gaussian 16 Rev. A.03; Wallingford, CT, 2016.

[16] M. J. Abraham, T. Murtola, R. Schulz, S. Páll, J. C. Smith, B. Hess, E. Lindahl. *SoftwareX*. **2015**, 1-2, 19-25.

[17] U. Essmann, L. Perera, M. L. Berkowitz, T. Darden, H. Lee, L. G. Pedersen. *J. Chem. Phys*. **1995**, 103, 8577-8593.

[18] P. J. Steinbach, B. R. Brooks. *J. Comput. Chem.* **1994**, 15, 667-683.

[19] B. Hess, H. Bekker, H. J. Berendsen, J. G. E. M. Fraaije. *J. Comput. Chem.* **1997**, 18, 1463-

1472.

[20] R. W. Hockney, S. P. Goel, J. W. Eastwood. *J. Comput. Phys.* **1974**, 14, 148-158.

[21] G. Bussi, D. Donadio, M. Parrinello. *J. Chem. Phys.* **2007**, 126, 014101.

[22] C. Lefebvre, G. Rubez, H. Khartabil, J. C. Boisson, J. C. Garcia, E. Henon. *Phys. Chem. Chem. Phys.,* **2017**, 19, 17928-17936.

[23] T. Lu. *J. Chem. Phys.*, **2024**, 161, 082503.

[24] W. Humphrey, A. Dalke, K. Schulten. *J. Mol. Graph.* **1996**, 14, 33-38.
